# Epidemic of influenza A(H1N1)pdm09 analyzed by full genome sequences and the first case of oseltamivir-resistant strain in Myanmar 2017

**DOI:** 10.1371/journal.pone.0229601

**Published:** 2020-03-04

**Authors:** Su Mon Kyaw Win, Reiko Saito, Nay Chi Win, Di Ja Lasham, Yadanar Kyaw, Nay Lin, Khin Nyo Thein, Irina Chon, Takashi Odagiri, Win Thein, Latt Latt Kyaw, Ommar Swe Tin, Akihiko Saitoh, Tsutomu Tamura, Chika Hirokawa, Yuko Uchida, Takehiko Saito, Shinji Watanabe, Takato Odagiri, Kazuhiro Kamata, Hidekazu Osada, Clyde Dapat, Hisami Watanabe, Htay Htay Tin

**Affiliations:** 1 Infectious Diseases Research Center of Niigata University in Myanmar (IDRC), Yangon, Yangon Region, Myanmar; 2 Division of International Health, Graduate School of Medical and Dental Sciences, Niigata University, Niigata, Niigata, Japan; 3 Respiratory Medicine Department, Thingangyun Sanpya General Hospital, Yangon, Yangon Region, Myanmar; 4 Clinical Laboratory, Microbiology Section, Pyinmana General Hospital, Pyinmana Township, Nay Pyi Taw, Myanmar; 5 Pediatric Ward 1, Yankin Children Hospital, Yangon, Yangon Region, Myanmar; 6 Department of Microbiology, Infectious diseases and Immunology, Iwate Medical University, Morioka, Iwate, Japan; 7 National Health Laboratory, Department of Medical Services, Ministry of Health and Sports, Yangon, Yangon Region, Myanmar; 8 Department of Pediatrics, Graduate School of Medical and Dental Sciences, Niigata University, Niigata, Niigata, Japan; 9 Division of Virology, Niigata Prefectural Institute of Public Health and Environmental Sciences, Niigata, Niigata, Japan; 10 Division of Transboundary Animal Disease, National Agriculture and Food Research Organization (NARO), Tsukuba, Ibaraki, Japan; 11 Laboratory of Influenza Virus Surveillance, Influenza Research Center, National Institute of Infectious Diseases, Sinjuku-ku, Tokyo, Japan; 12 Institute of Medicine and Dentistry, Niigata University, Niigata, Japan; 13 Department of Virology, Tohoku University Graduate School of Medicine, Sendai, Miyagi, Japan; Defense Threat Reduction Agency, UNITED STATES

## Abstract

A community outbreak of human influenza A(H1N1)pdm09 virus strains was observed in Myanmar in 2017. We investigated the circulation patterns, antigenicity, and drug resistance of 2017 influenza A(H1N1)pdm09 viruses from Myanmar and characterized the full genome of influenza virus strains in Myanmar from in-patients and out-patients to assess the pathogenicity of the viruses. Nasopharyngeal swabs were collected from out-patients and in-patients with acute respiratory tract infections in Yangon and Pyinmana City in Myanmar during January-December 2017. A total of 215 out-patients and 18 in-patients infected with A(H1N1)pdm09 were detected by virus isolation and real-time RT-PCR. Among the positive patients, 90.6% were less than 14 years old. Hemagglutination inhibition (HI) antibody titers against A(H1N1)pdm09 viruses in Myanmar were similar to the recommended Japanese influenza vaccine strain for 2017–2018 seasons (A/Singapore/GP1908/2015) and WHO recommended 2017 southern hemisphere vaccine component (A/Michigan/45/2015). Phylogenetic analysis of the hemagglutinin sequence showed that the Myanmar strains belonged to the genetic subclade 6B.1, possessing mutations of S162N and S164T at potential antigenic sites. However, the amino acid mutation at position 222, which may enhance the severity of disease and mortality, was not found. One case with no prior history of oseltamivir treatment possessed H275Y mutated virus in neuraminidase (NA), which confers resistance to oseltamivir and peramivir with elevated IC_50_ values. The full genome sequence of Myanmar strains showed no difference between samples from in-patients and out-patients, suggesting no additional viral mutations associated with patient severity. Several amino acid changes were observed in PB2, PB1, and M2 of Myanmar strains when compared to the vaccine strain and other Asian strains. However, no mutations associated with pathogenicity were found in the Myanmar strains, suggesting that viral factors cannot explain the underlying reasons of the massive outbreak in Myanmar. This study reported the first detection of an oseltamivir-resistant influenza virus in Myanmar, highlighting the importance of continuous antiviral monitoring and genetic characterization of the influenza virus in Myanmar.

## Introduction

Influenza outbreaks occur mainly during winter in temperate areas of the northern and southern hemispheres; however, influenza outbreaks in tropical areas around the equator may occur at any time of the year [1]. Influenza is not only responsible for local epidemics annually but also global pandemics. Since the 20^th^ century, there have been four flu pandemics: 1918 H1N1 Spanish flu [2], 1957 H2N2 Asian flu, 1968 H3N2 Hong Kong flu, and 2009 H1N1 swine flu. The 1918 H1N1 Spanish flu, which killed more than 50 million people worldwide, was the most severe pandemic [3].

Influenza virus is a highly infectious respiratory pathogen manifesting a significant threat to global public health [1]. Influenza A(H1N1)pdm09 virus, which emerged in 2009 and caused a global influenza pandemic, is now a seasonal influenza virus that co-circulates with another seasonal influenza (H3N2) and influenza B viruses. Worldwide circulation of A(H1N1)pdm09 virus has raised concerns about genotypic diversity that enhances virus transmissibility and pathogenicity and affects vaccine efficacy [4].

In 2008, Myanmar established a National Influenza Centre (NIC) at the National Health Laboratory in Yangon, which has been sharing data with the WHO by sending influenza isolates twice annually to the WHO collaborating center for new vaccine development, influenza genotyping, antiviral susceptibility, monitoring Influenza-like illness (ILI), and Severe Acute Respiratory Infection (SARI) surveillance. However, influenza surveillance efforts in Myanmar are limited. In 2017, the ILI and SARI surveillance system in Myanmar was strengthened by placing the sentinel sites approach, which was reinforced by the Ministry of Health and Sports of Myanmar (MoHS) with the support of WHO [5].

In Myanmar, the first pandemic influenza A(H1N1) 2009 case was detected in June 2009, with no reported fatal cases. Since then, it has been co-circulating with other seasonal influenza viruses in the country. Previously, we reported the epidemiology and genetic characterization of influenza virus A and B circulating in Myanmar [6, 7] and characterized the drug-susceptibility of seasonal and pandemic influenza A(H1N1) viruses in Myanmar in 2008 and 2009 [8]. Only 16 sporadic cases of A(H1N1)pdm09 were detected in 2009, and genotyping of these viruses showed no mutations in neuraminidase (NA), which indicated susceptibility to oseltamivir [8].

In July 2017, severe cases of influenza A(H1N1)pdm09 were detected in Myanmar, and the MoHS declared a high alert to mitigate the influenza outbreaks in the country. The overall numbers of severe pneumonia cases and fatal cases reached 1198 and 38, respectively [9]. This 2017 outbreak had a significant impact on the society of Myanmar. Eventually, the outbreak was successfully controlled in October 2017 by implementing and strengthening active influenza surveillance through early and timely detection of cases and the distribution of clinical management guidelines at hospitals (S1 File).

In this study, we investigated the circulation patterns, distribution of influenza subtypes, antigenic and genetic characterization of influenza A(H1N1)pdm09 virus strains and assessed their susceptibility to neuraminidase inhibitors (NAIs) in out-patients and in-patients in Myanmar during the 2017 influenza season.

## Materials and methods

### Study population

Out-patients with influenza-like illness symptoms, who visited two surveillance site hospitals, Thingangyun Sanpya General Hospital in Yangon and Pyinmana 200 Bedded General Hospital in Pyinmana, and in-patients with acute respiratory infections, who were admitted to Yankin Children Hospital (YKCH) in Yangon, were enrolled in the study between January and December 2017. The inclusion criteria for out-patients were sudden onset of fever (> 37.8 °C) with more than two of the following symptoms: cough, rhinorrhea, myalgia, arthralgia, and diarrhea; the exclusion criteria were suspected cases of digestive tract infections and chronic respiratory infections such as tuberculosis. The inclusion criteria for in-patients were history or measured fever of ≥ 38 °C, cough, onset within the last 10 days, difficulty in breathing with chest in-drawing for children under 5 years of age, and requirement for hospitalization, while the exclusion criteria were TB infection, chronic respiratory diseases, patients with tracheostomy, immunosuppressive status (e.g., HIV infection, chemotherapy), cystic fibrosis, and cancer. Written informed consents were obtained from patients, and clinical information such as name, age, sex, address, date of symptom onset, date of clinic visit, flu vaccination history, antiviral drug medication history, and symptoms were recorded in registration sheets before specimen collection.

### Sample collection

Two nasopharyngeal swabs were taken from each suspected patient. One swab was used for testing with a rapid test kit (Quick Navi-Flu+RSV, Denka Seiken Co. Ltd., Tokyo, Japan) to screen influenza virus at the sample collection sites. The other swab was stored in a viral transport media for further analysis at the National Health Laboratory (NHL), Myanmar, and Division of International Health, Niigata University, Japan.

### Virus isolation

The positive samples for influenza A and B, detected by real-time RT-PCR analysis, were inoculated in confluent Madin-Darby canine kidney (MDCK) cells to isolate the influenza viruses. The cell culture tubes were then incubated at 34 °C with 5% CO_2_ and observed daily for 5 days until the second passage to detect the specific cytopathic effect.

### RNA extraction and real-time RT-PCR

Viral RNA was extracted from 140 μL of nasopharyngeal swab supernatant of the original clinical sample for initial typing and from viral isolate for subtyping/lineage detection of influenza A or B using QIAamp Viral RNA mini Kit (QIAGEN, Hilden, Germany) following manufacturer’s instructions. Real-time PCR using QuantiTect Probe RT-PCR kit (QIAGEN) was performed according to the protocols provided by the National Institute of Infectious Diseases in Tokyo, Japan [10] to screen influenza viruses from original clinical specimens for initial typing as Type A or B. Subtyping PCR for A(H1N1)pdm09 or A(H3N2) and differentiation for B/Victoria or B/Yamagata lineages was performed at NHL for the virus isolates using specific primers and probes, respectively [10].

### Antigenic characterization

The antigenicity of selected A(H1N1)pdm09 isolates was characterized by hemagglutination inhibition (HI) assay following the WHO manual using guinea pig red blood cells and commercially available vaccine strain antisera for 2017–2018 seasons in Japan (A/Singapore/GP1908/2015, A/Michigan/45/2015-like strain) at the Department of Virology, Niigata Prefectural Institute of Public Health and Environmental Sciences, Niigata, Japan [11].

### Cycling probe real-time PCR

RNA was extracted from A(H1N1)pdm09 positive isolates using Extragen II kits (Toso, Tokyo, Japan). Complementary DNA was synthesized using uni12 primer for generic influenza A amplification, as described previously [12]. Cycling probe real-time PCR for the mutation on NA gene that confers resistance to oseltamivir was performed on the isolates using fluorescent-labeled chimeric RNA-DNA probes, RNase H, and the commercially available cycling probe real-time PCR kit, CycleavePCRCore kit (TaKaRa Bio Inc., Ohtsu, Japan), at the Division of International Health, Niigata University, Japan. These cycling probes were labeled with either 6-carboxyfluorescein (FAM) or 6-carboxy-X-rhodamine (ROX), and the PCR reaction was prepared according to the manufacturer’s instructions. This assay utilizes single nucleotide polymorphisms (SNP) for detecting oseltamivir-sensitive (H275) and oseltamivir-resistant (H275Y) viruses based on the reaction curves of FAM (H275) or ROX (H275Y) [12].

### Neuraminidase inhibitors susceptibility assay

The selected influenza A(H1N1)pdm09 viruses were tested using fluorescence-based inhibition assay (NA inhibition assay) against four kinds of neuraminidase inhibitors, oseltamivir (Sequoia Research Products Ltd., Pangbourne, UK), zanamivir (Sequoia Research Products Ltd.), peramivir (Shionogi & Co., Ltd., Osaka, Japan), and laninamivir (Daiichi Sankyo Co., Ltd., Tokyo, Japan). Susceptibility of viruses from culture supernatants of infected MDCK cells were used to test the inhibitory effect of each NA inhibitor (NAI). Prior to NAI assay, NAIs were diluted to a final concentration range of 0.005–1250 nM. Each influenza virus isolate was diluted to a final concentration of 25,000 fluorescence units to obtain a dilution in the linear range of the NA activity curve. Twenty-five microliters of each diluted NAI was added to each well of a microtiter plate, followed by the addition of 25 μL of each virus dilution [13]. The plates were incubated at 37 °C for 30 min. Fifty microliters of 2′-(4-Methylumbelliferyl)-α-D-N-acetylneuraminic acid (MUNANA) substrate was added to each well at a final concentration of 25 μM, and then, the plates were incubated at 37 °C for 60 min. The reaction was finally stopped by adding 260 μL of 200 mM sodium carbonate to each well. The fluorescence of the released 4-methyl umbelliferone (4-MUNANA) was measured in a microplate reader TriStar LB942 (Berthold Japan K.K., Tokyo, Japan) using an excitation wavelength of 360 nm and an emission wavelength of 460 nm. Inhibitory effect was expressed as the IC_50_ calculated using XLfit software (IDBS, Surrey, UK). The strain was assessed as resistant if the IC_50_ value against one of four drugs is 100-fold elevated for influenza A [14]. Drug susceptibility assay was performed at Niigata University (Niigata, Japan).

### Full genome sequencing using next-generation sequencing

The eight viral RNA segments for the selected Myanmar A(H1N1)pdm09 viruses were sequenced using next-generation sequencing to obtain the whole viral genome. Briefly, a cDNA library was prepared using random hexamers and NEBNext UltraTM RNA Library Prep kit (NEB) according to the manufacturer’s instructions, and sequenced using a MiSeq second-generation sequencer (Illumina) with Reagent Kit v2. The genomic sequences of the isolates were determined using FluGAS software (version 1.0.0, World Fusion, Tokyo, Japan), which mapped the output pair-end reads to reference sequences of each gene segment selected from the Influenza Virus Database in the National Center for Biotechnology Information Search database (NCBI) by the FluGAS algorithm. A consensus sequence was constructed when coverage was higher than three at each site and adopted a single nucleotide in at least 51% or more coverage, whereas mixed-base codes were adopted when multiple bases shared more than 15% out of the total coverage at the site. In this study, all of the consensus sequences subjected for phylogenetic analysis had more than 10 coverage at any site. The variant frequency at position 275 of the amino acid sequence in NA, which confers oseltamivir-resistance, was calculated using Genome Workbench (QIAGEN, Duesseldorf, Germany) software. Next-generation sequencing was performed at the National Institute of Animal Health, Tsukuba city, Japan.

### Phylogenetic analysis

In addition to Myanmar strains, A(H1N1)pdm09 collected from various prefectures (Okinawa, Nagasaki, Gunma, Shizuoka, Kyoto, Nara, and Hokkaido prefectures) in Japan as part of the influenza investigation conducted by Niigata University during the 2017–2018 season was also sequenced by Sanger Method [15]. All the sequences used in this study were registered with the Global Initiative on Sharing All Influenza Data (GISAID) (S1 and S2 Tables). Reference strains such as the influenza vaccine strains for the 2017–2018 Japanese vaccine, A/Singapore/GP1908/2015, and the 2017 southern hemisphere vaccine, A/Michigan/45/2015, as well as strains collected in India during May and August of 2017 (GISAID Isolate ID: EPI_ISL_281596, EPI_ISL_281597, EPI_ISL_281600, EPI_ISL_281601, EPI_ISL_281602, EPI_ISL_281603, EPI_ISL_281604, and EPI_ISL_282867) were downloaded from GISAID. India is specifically included in this analysis because A(H1N1)pdm09 outbreaks due to Clade 6B and 6B.1 viruses were repeatedly reported in India during 2015–2017, thus suggesting the possibility that the Myanmar strains may have originated in India [16–20]. Phylogenetic analysis was performed using the Hasegawa-Kishino-Yano model with discrete Gamma distribution (HKY+G) and the General Time Reversible model with discrete Gamma distribution incorporating Invariant sites (GTR + G + I) as the best-fit nucleotide substitution models for the HA and NA datasets, respectively, as implemented in the MEGA 6.0 software (Molecular Evolutionary Genetics Analysis) [21]. Best fitting trees for the HA and NA genes were constructed by the maximum likelihood method and bootstrap analysis of 1,000 replicates. Deduced amino acid sequences were analyzed, and amino acid changes were compared with A/California/07/2009 (H1N1pdm09) (GISAID Isolate ID: EPI_ISL_227813).

The six internal genes of A(H1N1)pdm09 from Myanmar and India were aligned by each segment using BioEdit software. The FASTA files of the multiple alignment of each segment were uploaded to the FluSurver (https://flusurver.bii.a-star.edu.sg/) to find any amino acid mutations compared to the vaccine strain, A/Michigan/45/2015, a WHO-recommended A(H1N1)pdm09 strain for the Southern hemisphere in 2017 [22], and to search for mutations that have significant functional or pathological implications.

### Ethical statement

This study was approved by the Niigata University Ethical Committee (2533) and the Ethical Review Committee in the Department of Medical Research, Ministry of Health and Sports, Myanmar (016516). Written consent was obtained from all study participants before sample collection.

## Results

### Number of samples and detection of influenza virus types and subtypes

From January to December of 2017, we collected a total of 328 nasopharyngeal swab samples (267 samples from Yangon study site and 61 samples from Pyinmana study site of Myanmar) from out-patients presenting influenza-like illness symptoms and 288 nasopharyngeal swab samples (from Yangon study site of Myanmar) from in-patients presenting acute respiratory tract infections, and tested the samples for the presence of influenza virus. Rapid test kit assay indicated that a total of 280 samples (45.5%) were positive for the influenza virus. All 616 nasopharyngeal swab samples were also screened by real-time PCR assay. Influenza A(H1N1)pdm09 viruses represented the majority of circulating influenza viruses during 2017 (233, 37.8%) (Table 1).

**Table 1:** Number of samples and fraction of influenza virus-positive tests based on real-time PCR analysis in Yangon and Pyinmana study sites, Myanmar, 2017.

	Out-patients				In-patients		Total(N = 616)	
	Yangon (N = 267)		Pyinmana (N = 61)		Yangon (N = 288)			
	N	(%)*	N	(%)*	N	(%)*	N	(%)*
**Rapid Test (+)**	215	(80.5)	46	(75.4)	19	(6.6)	280	(45.5)
Flu A	206	(77.1)	46	(75.4)	19	(6.6)	271	(44.0)
Flu B	9	(3.4)	0	(0)	0	(0)	9	(1.5)
**Real Time PCR (+)**	213	(79.8)	48	(78.7)	21	(7.3)	282	(45.8)
A(H1N1)pdm09	169	(63.3)	46	(75.4)	18	(6.3)	233	(37.8)
A(H3N2)	34	(12.7)	2	(3.3)	3	(1)	39	(6.3)
B/Victoria	0	(0)	0	(0)	0	(0)	0	(0)
B/Yamagata	10	(3.8)	0	(0)	0	(0)	10	(1.6)

(+): positive

* Percentage are shown for detection (influenza type A, B, subtype A and lineage B) on total collected samples

### Demographic and baseline clinical characteristics of influenza A(H1N1)pdm09 patients

In 2017, the median age of the influenza A(H1N1)pdm09 positive out-patients was 3.9 years in Yangon and 16.7 years in Pyinmana, respectively, and 2.2 years in the in-patient study. The differences detected between the median age of out-patients in Yangon and Pyinmana study sites was because the patients who were included in the Pyinmana study site were widely distributed in various age groups, from infants to adults (3 months to 56 years), and majority of the patients who visited the clinic were children less than 5 years old in Yangon for both in out-patients and in-patients groups (Table 2). All patients enrolled in this study did not receive any influenza vaccines. Overall the most commonly reported presenting symptoms of the influenza A(H1N1)pdm09-positive out-patients and in-patients were fever, cough, and rhinorrhea. Additionally, the majority of hospitalized patients had a history of dyspnea and wheezing on admission, reflecting the severity of the in-patients (Table 2).

**Table 2:** Demographic and baseline clinical characteristics of A(H1N1)pdm09 patients, Myanmar, 2017.

Variables	Out-patients		In-patients
Study site	Yangon (N = 169)	Pyinmana (N = 46)	Yangon (N = 18)
**Age**			
median [range]	3.9 [0.5–28.0]	16.7 [0.25–56.0]	2.2 [0.3–5.5]
**Age groups**	**n (%)**	**n (%)**	**n (%)**
<5 yrs	116 (68.6%)	9 (19.6%)	16 (88.9%)
5–14 yrs	50 (29.6%)	18 (39.1%)	2 (11.1%)
15–29 yrs	3 (1.8%)	11 (23.9%)	0 (0.0%)
30–64 yrs	0 (0.0%)	8 (17.4%)	0 (0.0%)
> = 65 yrs	0 (0.0%)	0 (0.0%)	0 (0.0%)
**Gender**	**n (%)**	**n (%)**	**n (%)**
Female	62 (36.7%)	14 (30.4%)	8 (44.4%)
Male	107 (63.3%)	32 (69.6%)	10 (55.6%)
**Symptoms**	**Presence (%)**	**Presence (%)**	**Presence (%)**
Fever	139 (82.2%)	44 (95.7%)	14 (77.8%)
Cough	143 (84.6%)	37 (80.4%)	18 (100%)
Wheezing	0 (0.0%)	4 (8.7%)	12 (66.7%)
Rhinorrhea	130 (76.9%)	43 (75.4%)	13 (72.2%)
Dyspnea	0 (0.0%)	0 (0.0%)	14 (77.8%)
Headache	0 (0.0%)	21 (45.7%)	NA
Myalgia	2 (1.2%)	28 (60.9%)	NA
Arthralgia	1 (0.6%)	28 (60.9%)	NA
Diarrhea	0 (0.0%)	5 (10.9%)	NA

n = number of patients that responded by “yes” or “no” for a given symptom.

NA—Not Applicable

**Fig 1:**
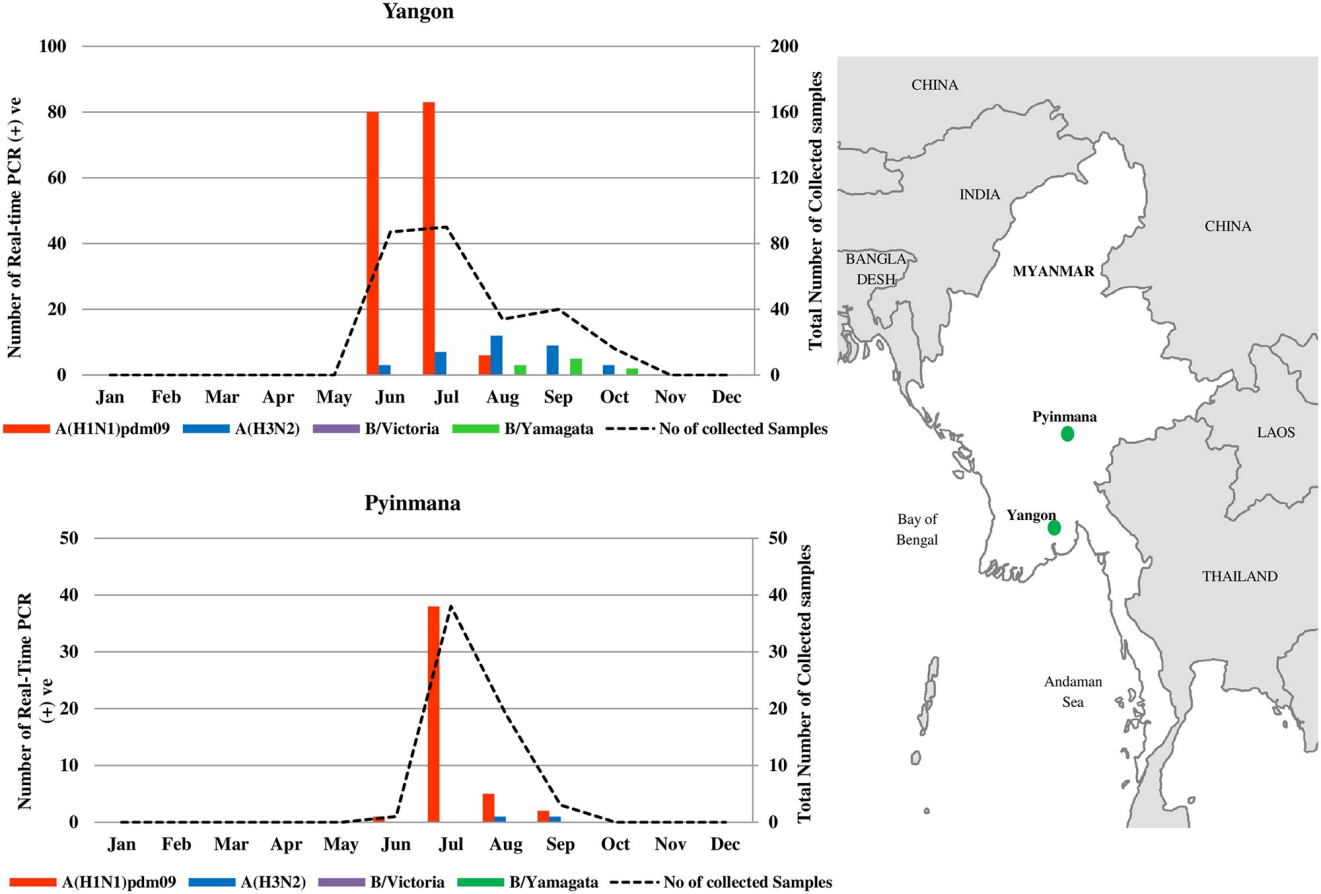
Monthly distribution of influenza virus by region in Myanmar during the 2017 influenza season and the location of sample collection sites. The total number of collected samples is shown in the black dashed line. Influenza A(H1N1) pdm09 is shown as red bar; A(H3N2), blue bar; B/Victoria, purple bar and B/Yamagata, green bar. The seasonal peak of A(H1N1)pdm09 was observed in June-July, during the rainy season, and influenza A(H1N1)pdm09 was the predominant subtype in Myanmar during the 2017 influenza season. The map of Myanmar and the green circles represent the two study sites, Yangon and Pyinmana. This map is generated by the software ArcMap 10.0 (ESRI Japan Corporation, Tokyo, Japan). Boundaries are generated by using a world basemap supplied by ESRI Japan Corporation.

During 2017, the monthly distribution of influenza virus-positive, as detected by real-time PCR analysis, revealed that the influenza season in Myanmar started between June and October. Influenza A(H1N1)pdm09 was the predominant subtype in the two study sites, Yangon (169/267, 63.3%) and Pyinmana (46/61, 75.4%) (Table 1). The seasonal peak of A(H1N1)pdm09 was observed in July, during the rainy season of Myanmar, in both Yangon and Pyinmana study sites (Fig 1). Influenza A(H3N2) circulated throughout the season (from June to October) at low levels in both Yangon and Pyinmana. Influenza B viruses detected in Yangon belonged to the B/Yamagata lineage and were observed from August to October, while influenza B was not detected in Pyinmana (Fig 1).

### Antigenic characterization of influenza A(H1N1)pdm09 viruses

The twelve selected influenza A(H1N1)pdm09 isolates were characterized by HI assay to determine the antigenicity of the influenza virus strains circulating during the outbreak season using post-infection rabbit antisera raised against the vaccine virus, A/Singapore/GP1908/2015, that was used for the Japanese influenza vaccine strain for the 2017–2018 seasons [23]. A/Singapore/GP1908/2015 is antigenically similar to A/Michigan/45/2005, a WHO selected influenza vaccine component for the 2017 southern hemisphere [22, 24]. All twelve test viruses were well recognized by the antiserum raised against the vaccine virus, A/Singapore/GP1908/2015, with titers were 2- and 4-folds higher than the homologous titer (Table 3). Myanmar influenza A(H1N1)pdm09 viruses were antigenically similar to the vaccine virus A/Singapore/GP1908/2015.

**Table 3:** Antigenic analysis of selected influenza A(H1N1)pdm09 viruses from Myanmar 2017.

Viruses		Hemagglutination inhibition (HI) titer	Fold-increase
		Post-infection rabbit antisera	
		A/Singapore/GP1908/2015	
REFERENCE VIRUSES	examination date		
A/Singapore/GP1908/2015*	5 Dec 2017	2560	
TEST VIRUSES			
A/Myanmar/17M007/2017		10240	4
A/Myanmar/17M012/2017		5120	2
A/Myanmar/17M015/2017		10240	4
A/Myanmar/17M023/2017		5120	2
A/Myanmar/17M025/2017		10240	4
A/Myanmar/17M062/2017		5120	2
A/Myanmar/17M064/2017		10240	4
A/Myanmar/17M083/2017		10240	4
A/Myanmar/17M108/2017		5120	2
A/Myanmar/17M109/2017		5120	2
A/Myanmar/17M115/2017		5120	2
A/Myanmar/17M204/2017		10240	4

*Influenza vaccine strain used in Japan for the 2017/18 seasons.

### Drug resistant influenza A(H1N1)pdm09 virus

Of the 233 influenza A(H1N1)pdm09 viruses from all the study sites, one isolate (0.4%) had the oseltamivir-resistance with H275Y mutation in the NA protein, as analyzed by cycling probe real-time PCR. The isolate originated from an out-patient, who was a 28 years old male with an onset date on 7 August 2017. He visited the medical institution in Pyinmana with a body temperature of 37.2 °C, headache, runny nose, and myalgia. He did not receive oseltamivir treatment prior to and after the medical visit. His precise illness course is unknown, but he recovered without complications. The epidemiological link of the H275Y mutant virus is unknown. Next-generation sequencing analysis revealed that the variant frequency of oseltamivir-resistance histidine to tyrosine amino acid substitution at position 275 in the NA gene of this mutant virus was 99.8% (495 reads/496 total reads). All the other A(H1N1)pdm09 isolates from Yangon and Pyinmana study sites did not show H275Y mutation in NA (Table 4).

**Table 4:** Frequency of H275Y mutated A(H1N1)pdm09 virus in 2017 influenza season using cycling probe real-time PCR method.

Location		H275Y (N)	A(H1N1)pdm09 (N)	%
Out-patients	Yangon	0	169	0.0
	Pyinmana	1	46	2.2
In-patients	Yangon	0	18	0.0
Total		1	233	0.4

*In vitro* fluorescence-based NAI assay performed on the selected influenza A(H1N1)pdm09 isolates (12 isolates from out-patients and 12 from in-patients) revealed that the H275Y mutant A(H1N1)pdm09 virus has elevated IC_50_ value for oseltamivir (301.5 nM with 198-fold increase) and peramivir (21.9 nM with 274-fold increase) but not for zanamivir (0.58 nM with 0.9-fold difference) and laninamivir (0.72 nM with 2.7-fold difference), when compared to the IC_50_ value of the reference drug-sensitive strain A/Perth/265/2009 (275H), indicating resistance to oseltamivir and peramivir with highly reduced inhibition. All of the remaining A(H1N1)pdm09 viruses were sensitive to the four neuraminidase inhibitors with IC_50_ values of less than 10-fold difference compared to the reference virus (Table 5).

**Table 5:** IC_50_ values of influenza A(H1N1)pdm09 viruses in Myanmar, 2017.

Strain Name	Status	Drug susceptibility related mutation in NA	IC_50_^a^ [nM](fold difference)^b^			
			Oseltamivir	Peramivir	Zanamivir	Laninamivir
A/Myanmar/17M012/2017	Out		0.50 (0.3)	0.07 (0.9)	0.11 (0.2)	0.07 (0.3)
A/Myanmar/17M015/2017	Out		0.72 (0.5)	0.12 (1.5)	0.12 (0.2)	0.09 (0.3)
A/Myanmar/17M023/2017	Out		0.50 (0.3)	0.07 (0.9)	0.11 (0.2)	0.08 (0.3)
A/Myanmar/17M025/2017	Out		0.64 (0.4)	0.08 (1)	0.13 (0.2)	0.09 (0.3)
A/Myanmar/17M062/2017	Out		0.49 (0.3)	0.07 (0.9)	0.11 (0.2)	0.09 (0.3)
A/Myanmar/17M064/2017	Out		0.50 (0.3)	0.07 (0.9)	0.10 (0.2)	0.08 (0.3)
A/Myanmar/17M083/2017	Out		0.58 (0.4)	0.08 (1)	0.12 (0.2)	0.09 (0.3)
A/Myanmar/17M108/2017	Out		0.68 (0.5)	0.09 (1.1)	0.17 (0.3)	0.11 (0.4)
A/Myanmar/17M109/2017	Out		0.41 (0.3)	0.08 (1)	0.10 (0.2)	0.09 (0.3)
A/Myanmar/17M115/2017	Out		0.56 (0.4)	0.08 (1)	0.11 (0.2)	0.09 (0.3)
A/Myanmar/17M204/2017	Out		0.46 (0.3)	0.07 (0.9)	0.37 (0.6)	0.30 (1.1)
A/Myanmar/17M307/2017	Out	H275Y	301.5 (198)	21.9 (274)	0.58 (0.9)	0.72 (2.7)
A/Myanmar/17MP001/2017	In		0.56 (0.4)	0.08 (1)	0.34 (0.6)	0.32 (1.2)
A/Myanmar/17MP002/2017	In		0.55 (0.4)	0.07 (0.9)	0.46 (0.7)	0.31 (1.2)
A/Myanmar/17MP003/2017	In		0.55 (0.4)	0.08 (1)	0.46 (0.7)	0.33 (1.2)
A/Myanmar/17MP004/2017	In		0.57 (0.4)	0.07 (0.9)	0.38 (0.6)	0.29 (1.1)
A/Myanmar/17MP005/2017	In		0.64 (0.4)	0.07 (0.9)	0.40 (0.7)	0.30 (1.1)
A/Myanmar/17MP009/2017	In		0.72 (0.5)	0.10 (1.3)	0.46 (0.7)	0.32 (1.2)
A/Myanmar/17MP013/2017	In		0.56 (0.4)	0.05 (0.6)	0.77 (1.2)	0.29 (1.1)
A/Myanmar/17MP014/2017	In		0.56 (0.4)	0.09 (1.1)	0.39 (0.6)	0.32 (1.2)
A/Myanmar/17MP015/2017	In		0.59 (0.4)	0.07 (0.9)	0.39 (0.6)	0.28 (1)
A/Myanmar/17MP018/2017	In		0.83 (0.6)	0.12 (1.5)	0.45 (0.7)	0.34 (1.3)
A/Myanmar/17MP019/2017	In		0.63 (0.4)	0.08 (1)	0.38 (0.6)	0.29 (1.1)
A/Myanmar/17MP021/2017	In		0.61 (0.4)	0.08 (1)	0.32 (0.5)	0.26 (1)

“Out” indicates out-patient and “In” indicates in-patient in the status

^a^ = Generated in fluorescent-based NI assay.

^b^ = Compared with the reference strain A/Perth/265/2009 (275H) with IC_50_ values for oseltamivir being 1.52nM, peramivir 0.08nM, zanamivir 0.62nM, laninamivir 0.27nM.

### Genetic characterization of HA and NA proteins of influenza A(H1N1)pdm09 viruses

**Fig 2:**
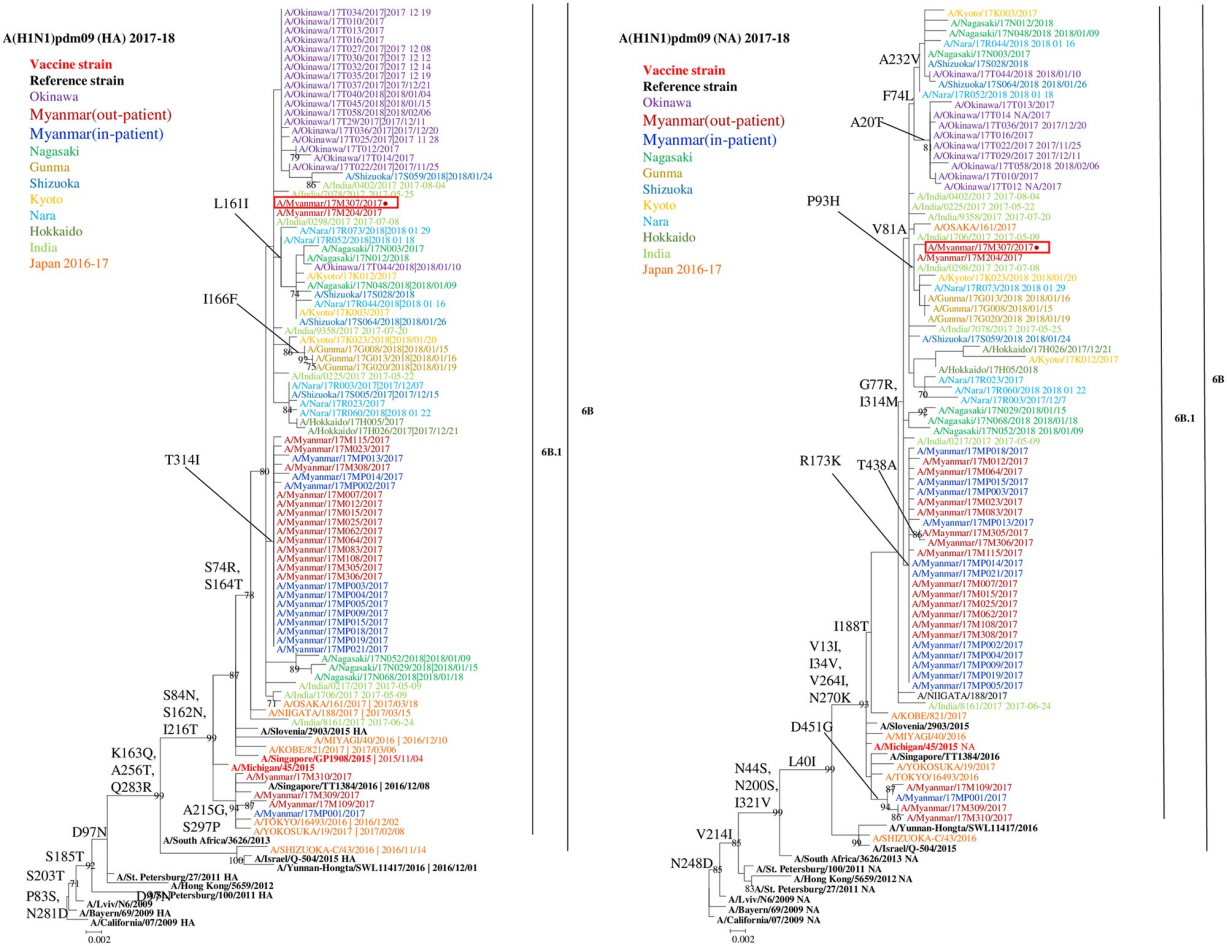
HA and NA phylogeny of influenza A(H1N1) 2009 pandemic isolates in Myanmar in 2017. Trees were constructed by the maximum likelihood method using MEGA software (version 6.06). Bootstrap value was determined for 1000 iterations; only values greater than 70% are shown. Myanmar out-patient is shown in red fonts, and in-patient is shown in dark blue fonts. The HA and NA sequences of Myanmar strains belonged to clade 6B.1. Red circle (●) represents oseltamivir- and peramivir-resistant strain exhibiting NA H275Y substitution. Amino acid changes were based on A/California/07/2009.

The HA and NA genes of influenza A(H1N1)pdm09 viruses in samples selected from 18 out-patients and 12 in-patients, including two cases with fatal outcomes, A/Myanmar/17MP009/2017 and A/Myanmar/17MP015/2017, were genetically characterized. To match the age group with in-patients, test samples of out-patients were randomly selected from children in the same age group (< 5 years old) for better comparison. We failed to sequence six out of 18 viruses from in-patients. All the analyzed Myanmar viruses belonged to genetic clade 6B.1 with amino acid substitutions of S84N, S162N, and I216T in HA [24] (Fig 2). The majority (26, 87%) of viruses possessed S164T in HA and formed a clade with the Indian 2017 and Japanese 2017–2018 strains. Among them, 13 viruses from out-patients and 11 viruses from in-patients possessed a T314I substitution in HA. It was a unique amino acid substitution common among the Myanmar sequences. Two Myanmar out-patient viruses (A/Myanmar/17M204/2017 and A/Myanmar/17M307/2017) did not belong to these clusters but were closely related to Indian strains in the same year, 2017. Three out-patient viruses (A/Myanmar/17M109/2017, A/Myanmar/17M309/2017, and A/Myanmar/17M310/2017) and one in-patient virus (A/Myanmar/17MP001/2017) shared A215G and S297P substitutions and formed a group with the Japanese strains in 2016 and 2017, having a bootstrap value of over 90%.

In the phylogeny of the NA gene, all the Myanmar viruses possessed V13I, I34V, V264I, and N270K substitutions and belonged to clade 6B.1. Around 60% of Myanmar out-patient viruses and all in-patient viruses shared the R173K amino acid substitution. Among the viruses from this cluster, two out-patient viruses possessed T438A substitution. H275Y mutation on neuraminidase was found in one A(H1N1)pdm09 strain (A/Myanmar/17M307/2017), which was from an out-patient with no prior history of anti-viral treatment. This resistant virus and another sensitive out-patient virus exhibited P93H substitution in NA. Three out-patient viruses and one in-patient virus exhibited the D451G substitution, which formed a small separate cluster as HA (Fig 2).

### Internal gene analysis of influenza A(H1N1)pdm09 viruses

We further characterized the amino acid substitutions in the remaining six segments of 25 Myanmar influenza A(H1N1)pdm09 strains, 13 out-patients including a case with NA/H275Y mutation and 12 in-patients, using next-generation sequencing. FluSurver research tool (https://flusurver.bii.a-star.edu.sg/) was used to screen the significant amino acid mutations in the 25 Myanmar and 8 Indian strains collected in 2017. The amino acid sequences of the six segments from the present study showed 99.0–99.8% identity with the reference strain A/Michigan/45/2015.

All Myanmar in-patient strains except one possessed the amino acid substitutions (as compared to A/Michigan/45/2015) at positions 66, 299, 398, and 453 in PB2, at positions 12 and 375 in PB1, at position 27 in M2, and at position 65 in NS1. All these mutations were frequently observed (10/13, 77%) in Myanmar out-patient strains. A/Myanmar/17MP009/2017 and A/Myanmar/17MP015/2017 strains from two fatal cases possessed the same amino acid mutations found in out-patients and in-patients who recovered from the disease (Table 6). A number of new mutations were observed in PB2 and PB1 in low frequencies, but none are indicated to have significant effect by FluSurver. Only sporadic mutations were found in PA and M1, NP and there was no common mutation in NS2 (S3 Table). In general, no difference was found between out-patients and in-patients. Differences in four amino acid mutations were found between Myanmar and Indian strains (Table 6); however, these mutations had no known functional difference except the mutation at position 27 in the M2 segment, which confers drug resistance to amantadine [25]. Of note, the first oseltamivir-resistant strain in Myanmar shared the same amino acid substitutions with the Indian strains (Table 6 and S3 Table).

**Table 6:** Abbreviated list of amino acid substitutions found in internal genes of influenza A(H1N1)pdm09 viruses collected from out-patients and in-patients in Myanmar compared with viruses from India in 2017.

Substitution	PB2				PB1		M2	NS1
Strain name	66	299	398	453	12	375	27^a^	65
A/Michigan/45/2015	I	R	T	P	I	S	V	M
**In-patients**								
A/Myanmar/17MP001/2017		K	I	T				V
A/Myanmar/17MP002/2017	T	K	I	T	V	N	A	V
A/Myanmar/17MP003/2017	T	K	I	T	V	N	A	V
A/Myanmar/17MP004/2017	T	K	I	T	V	N	A	V
A/Myanmar/17MP005/2017	T	K	I	T	V	N	A	V
A/Myanmar/17MP009/2017	T	K	I	T	V	N	A	V
A/Myanmar/17MP013/2017	T	K	I	T	V	N	A	V
A/Myanmar/17MP014/2017	T	K	I	T	V	N	A	V
A/Myanmar/17MP015/2017	T	K	I	T	V	N	A	V
A/Myanmar/17MP018/2017	T	K	I	T	V	N	A	V
A/Myanmar/17MP019/2017	T	K	I	T	V	N	A	V
A/Myanmar/17MP021/2017	T	K	I	T	V	N	A	V
**Out-patients**								
A/Myanmar/17M007/2017	T	K	I	T	V	N	A	V
A/Myanmar/17M012/2017	T	K	I	T	V	N	A	V
A/Myanmar/17M015/2017	T	K	I	T	V	N	A	V
A/Myanmar/17M023/2017	T	K	I	T	V	N	A	V
A/Myanmar/17M025/2017	T	K	I	T	V	N	A	V
A/Myanmar/17M062/2017	T	K	I	T	V	N	A	V
A/Myanmar/17M064/2017	T	K	I	T	V	N	A	V
A/Myanmar/17M083/2017	T	K	I	T	V	N	A	V
A/Myanmar/17M108/2017	T	K	I	T	V	N	A	V
A/Myanmar/17M109/2017		K	I	T		N		V
A/Myanmar/17M115/2017	T	K	I	T	V	N	A	V
A/Myanmar/17M204/2017			I	T				V
A/Myanmar/17M307/2017		K	I	T				V
**India**								
A/India/0217/2017		K	I	T				V
A/India/0225/2017		K	I	T				V
A/India/0298/2017		K	I	T				V
A/India/0402/2017		K	I	T				V
A/India/1706/2017		K	I	T				V
A/India/7078/2017		K	I	T				V
A/India/8161/2017		K	I	T				V
A/India/9358/2017		K	I	T				V

Analysis was performed by FluSurver (https://flusurver.bii.a-star.edu.sg/). A/Michigan/45/2015 was used as a reference automatically. A full list of amino acid substitutions can be found in the S3 Table.

^a^Reported to be related to drug resistance to amantadine.

## Discussion

A regional influenza outbreak with the major subtype of A(H1N1)pdm09 occurred in Myanmar from June to October in 2017, and an increased number of severe pneumonia cases were reported [9]. The viruses involved in this outbreak were genetically very similar to A(H1N1)pdm09 strains circulating in Asia and other countries during the same year. There were no differences in the genetic characteristics of A(H1N1)pdm09 influenza viruses collected from out-patients and in-patients, including cases with fatal outcomes. The antigenicity of the Myanmar viruses was similar to the vaccine strain. In addition, the first case of oseltamivir-resistant virus in Myanmar without prior oseltamivir treatment was detected in our study.

In Myanmar, the peak of influenza virus activity coincides with the rainy season. Myanmar has a tropical monsoon climate with three seasons: winter (November—February), summer (March—Mid May), and rainy (Mid May—October) seasons, based on the analysis of pressure, rainfall, and temperature [26]. The study showed that the circulation of influenza viruses peaked in July, which is consistent with previous studies showing that the influenza season in Myanmar peaked in July or August, during the rainy season [6–8]. This observation is also similar to the national surveillance data reported to the regional and global influenza surveillance platform (FluNET), which showed that the regional influenza outbreak started during the rainy season from July (week 29) to September (week 36), and influenza virus activity peaked in August with 369 flu-positive cases during the reported weeks 31–35 in 2017 [27]. Nationally, an increasing number of influenza cases was notified in July, and the large number of samples for influenza testing became a burden to NIC, which resulted in a restriction on sample handling in our study sites. Therefore, influenza detection was decreased in August at study sites compared to the national influenza peak period.

Antigenic analysis using the HI test showed that Myanmar strains in 2017 were similar to the vaccine strain A/Singapore/GP1908/2015, a vaccine component for the Japanese inactivated vaccine for the 2017/2018 season [22]. A/Singapore/GP1908/2015 belonged to 6B.1 clade and is antigenically similar to A/Michigan/45/2015, a vaccine component for the Southern hemisphere in 2017 [24, 28] and Northern hemisphere in 2017/2018 [29]. In this study, the HI results of the isolated viruses that reacted to the antisera raised against the vaccine strain had higher titer by 2–4 fold increase than the homologous titer of the vaccine strain virus. The higher HI titer of test viruses than vaccine viruses is often observed in the circulating strains, and it merely shows an antigenic match with the vaccine strains. Indeed, a recent report from the European Centre for Disease Prevention and Control shows that the HI titer of the circulating viruses was 2–4 fold higher, indicating a match with the vaccine and the reference viruses [30]. WHO changed the vaccine component from A/California/7/2009 to A/Michigan/45/2015 for the Southern hemisphere vaccine in 2017 because of the increasing number of clade 6B.1 viruses observed at the beginning of 2016. Clade 6B.1 viruses were reported to show similar antigenicity to ferret antisera against A/California/7/2009 when tested by the HI test, but they reacted poorly with pediatric, adult, and older adult pre- and post-vaccination sera [22]. Thus, WHO changed the vaccine component into A/Michigan/45/2015 from the 2017 southern hemisphere vaccine. In this outbreak, Myanmar experienced severe cases of influenza A/H1N1pdm09 in 2017; however, it turned out that these viruses matched with the vaccine strain.

According to the phylogenetic analysis, the 2017 Myanmar influenza A(H1N1)pdm09 strains belonged to clade 6B.1 and were closely related to the strains that circulated in India and other countries during the same period. India reported several outbreaks and severe cases caused by influenza A(H1N1)pdm09 of Clade 6B and 6B.1 during 2015–2017 [16–20, 31]. We suspect the possibility of transmission from India or other countries situated to the west of Myanmar because early cases of this outbreak were reported from Chin State, which shares its western border with Bangladesh. The analyzed strains in this study are genetically similar to the vaccine viruses, A/Michigan/45/2015 and A/Singapore/GP1908/2015. It was known that S74R, I295V mutations in HA protein were recently acquired by the virus; this mutation has high frequency of detection (66%) globally [17]. Moreover, about 32% of global strains had the S164T mutation, and it is reported as the most recent mutation observed among 2017 strains [17]. Similarly, these mutations were highly detected (87%) in Myanmar strains. In contrast to Myanmar strains, amino acid substitutions A73V, V321I, T508A, and I510T, were reported from Central India sequences in 2017 with low global occurrence [17].

Another known important amino acid change, D222N/G in HA, was not detected in viruses from Myanmar in-patients. This mutation affects virus tropism and enhances the severity of disease and mortality by assisting viral binding to α-2,3 receptors, which are mainly present in the lower respiratory tract with the occurrence of 0.4% frequency at global level [32–34]. In NA phylogeny, amino acid substitutions V13I, I34V, and I314M were found in all Myanmar strains. These substitutions were also detected in Indian and Nepalese A(H1N1)pdm09 outbreak strains in early 2015, which suggests the coevolution of HA and NA [16]. According to our investigation (unpublished data), influenza A(H3N2) and B viruses, which were the predominant circulating strains in Myanmar during 2016, could be a possible reason for this big outbreak of A(H1N1)pdm09 and it may be responsible for the reduced herd immunity against A(H1N1)pdm09, when the new 6B.1 clade virus was introduced in Myanmar in 2017.

Genetic characterization of six internal genes (PB2, PB1, PA, NP, MP, and NS) showed that there were no marked genetic changes related to increased pathogenicity. Several complex factors are associated with severe pneumonia: viral factors, such as mutations in the polymerase, HA, PB1-F2, polymerase acidic protein frameshift, and host factors, such as comorbidities, single-nucleotide polymorphism, inflammation, high cytokines and chemokines; bacterial factors, such as function of attachment, replication, and loss of repair can result in secondary bacterial infections [35]. No significant genetic differences were found between 13 viruses collected from out-patients and 12 viruses from in-patients. It was, thus, suggested that the severity of illness is not caused by the differences of virus strains between in-patients and out-patients but characteristics of influenza A(H1N1)pdm09 causing viral pneumonia [35–37]. In this study, internal genes of Indian strains and Myanmar strains were compared. The local circulation of A(H1N1)pdm09 was observed from 2016 to 2017 in India, Nepal, and Bangladesh prior to the outbreaks in Myanmar [38]. Among the four mutation differences between Myanmar and Indian strains, the only functional significance was at position 27 in the M2 segment that confers resistance to amantadine. Adamantane-resistant influenza variants have been circulating in the world for decades [25]. M2 gene mutations associated with resistance to amantadine were found in only about 1% of V27A mutations compared to 95% of S31N mutation [25]. Indeed, all of Myanmar and Indian strains in this study also possessed S31N mutation. It is highly presumable that strains from the two countries shared the same source of origin because Myanmar and Indian strains shared higher percentage of sequence identity. No additional viral genetic changes associated with disease severity were identified so far.

Here, we report the identification and detection of a H275Y mutation in NA that confer resistant to oseltamivir from an influenza A(H1N1)pdm09 isolate in community samples of Myanmar, which was detected for the first time in Myanmar; however, its detection rate was low at only 0.5%. In Myanmar, influenza A(H1N1)pdm09 was first detected in 2009 and the oseltamivir-resistant H275Y variant has not been detected in specimens, either from the community or hospitalized patients since then, although all isolates tested showed the S31N mutation in M2 that conferred resistance to amantadine [8]. Oseltamivir was not previously used for the treatment of influenza in Myanmar, but at the time of the outbreak in 2017, it was supplied by the WHO as an emergency control measure. The drug was administered to the admitted patients with severe cases of influenza infection (S1 File), but the number of prescribed courses for estimating the effect on the emergence of oseltamivir-resistant strain is not available. It was already reported worldwide that oseltamivir-resistant influenza A(H1N1)pdm09 virus infections were detected in community settings with no known exposure to oseltamivir and the resistant virus spread within the community even in the absence of drug-selective pressure [39, 40]. This fact suggests that there is a possibility of rapid transmission of resistant strains from patients, who were already treated with oseltamivir, in a short period after prescribing the antiviral drug in Myanmar. Moreover, this virus showed high reduced inhibition (HRI) with high fold-increase in IC_50_ (198 and 274) to two NAIs, oseltamivir and peramivir. Global analysis on the susceptibility of human influenza viruses to the NAI reported that the most common NA amino acid substitution was H275Y in A(H1N1)pdm09 viruses, which confers HRI by oseltamivir and peramivir [41] without affecting the susceptibility to zanamivir and laninamivir [42]. Despite the low prevalence of oseltamivir-resistance according to our study data, it highlights the necessity for continuous monitoring of the influenza virus and close surveillance of antiviral drug resistance in Myanmar.

There were no known fatal cases of influenza A(H1N1)pdm09 reported in Myanmar until 2017. Indeed 2 of our pediatric in-patients who underwent drug susceptibility and genetic sequencing were death cases. Moreover, detailed characterization of influenza A(H1N1)pdm09 viruses is very limited [8] and whole-genome sequencing of Myanmar influenza A(H1N1)pdm09 strains have not been characterized before our study.

Nonetheless, this study has several limitations. The samples tested herein were collected from two surveillance sites for out-patients and from one children hospital for in-patients. Due to the limitations of human resources in Myanmar, there was no hospital to collect samples from in-patients and out-patients in the same surveillance site within the study period. Therefore, we decided to select two hospitals for out-patients and one hospital for in-patients. Moreover, influenza-positive sample size of in-patients was quite small compared to out-patients even though we collected 288 in-patients. We detected the first oseltamivir-resistant strain in Myanmar, but the epidemiological link of the patient was unclear. Besides, we could analyze only two out-patients in Pyinmana by NGS. It would be more useful if we could increase the number of samples for sequencing for a more comprehensive analysis. Analysis of NGS data is still ongoing, and the results will be published elsewhere.

## Conclusion

In this study, we characterized the influenza A(H1N1)pdm09 strains that circulated in Myanmar during the 2017 outbreak and attempted to identify factors that are responsible for the virulence of the virus, which may cause the severity of influenza among in-patients by comparing with the out-patient strains. Although viral genome features responsible for the differential severity of influenza between in-patients and out-patients could not be thoroughly elucidated, we present the first-time characterization of Myanmar influenza viruses by the full genome sequencing. This study demonstrates the importance of continuing antiviral monitoring, epidemiological investigations, and genetic characterization in the data-limited country like Myanmar.

## Supporting information

S1 Table Details of whole genomes of Myanmar influenza A(H1N1)pdm09 used in this study. All the data are registered to the Global Initiative on Sharing All Influenza Data (GISAID).
(DOCX)

S2 Table Details of influenza A(H1N1)pdm09, HA and NA sequences used in the phylogenetic tree in this study. All the data are registered to the Global Initiative on Sharing All Influenza Data (GISAID).
(DOCX)

S3 Table Detail of amino acid substitutions found in PB2, PB1, PA, NP, MP, and NS protein of influenza A(H1N1)pdm09 viruses collected from out-patients and in-patients in Myanmar compared with viruses from India in 2017. (DOCX)

S1 File Influenza (H1N1)pdm infection. Management Protocol and Case Report Form. North Okkalapa General Hospital.
(PDF)
