# Supplementary material for: Epidemic of influenza A(H1N1)pdm09 analyzed by full genome sequences and the first case of oseltamivir-resistant strain in Myanmar 2017
Source: PLoS One. 2020 Mar 4;15(3):e0229601. doi: 10.1371/journal.pone.0229601 (PMC7055873; doi:10.1371/journal.pone.0229601)
Supplement: S1 Table — All the data are registered to the Global Initiative on Sharing All Influenza Data (GISAID). (DOCX) [file pone.0229601.s001.docx]

**S1 Table. Details of whole genomes of Myanmar influenza A(H1N1)pdm09 used in this study. All the data are registered at the Global Initiative on Sharing All Influenza Data (GISAID)**

| **Strain name** | **Collection date** | **Isolate ID** | **HA** | **NA** | **PB2** | **PB1** | **PA** | **NP** | **MP** | **NS** |
| --- | --- | --- | --- | --- | --- | --- | --- | --- | --- | --- |
| **A/Myanmar/17MP001/2017** | 2017-10-06 | **EPI_ISL_333328** | EPI1331960 | EPI1331961 | EPI1370008 | EPI1370007 | EPI1370006 | EPI1370004 | EPI1370003 | EPI1370005 |
| **A/Myanmar/17MP002/2017** | 2017-10-06 | **EPI_ISL_333329** | EPI1331962 | EPI1331963 | EPI1370009 | EPI1370010 | EPI1370011 | EPI1370012 | EPI1370013 | EPI1370014 |
| **A/Myanmar/17MP003/2017** | 2017-10-06 | **EPI_ISL_333335** | EPI1331978 | EPI1370015 | EPI1370016 | EPI1370017 | EPI1370018 | EPI1370019 | EPI1370020 | EPI1370021 |
| **A/Myanmar/17MP004/2017** | 2017-06-12 | **EPI_ISL_333354** | EPI1332040 | EPI1332041 | EPI1370022 | EPI1370023 | EPI1370024 | EPI1370025 | EPI1370026 | EPI1370027 |
| **A/Myanmar/17MP005/2017** | 2017-06-12 | **EPI_ISL_333356** | EPI1332043 | EPI1332044 | EPI1370302 | EPI1370303 | EPI1370304 | EPI1370305 | EPI1370306 | EPI1370307 |
| **A/Myanmar/17MP009/2017** | 2017-06-17 | **EPI_ISL_333357** | EPI1332045 | EPI1332046 | EPI1370308 | EPI1370309 | EPI1370310 | EPI1370311 | EPI1370312 | EPI1370313 |
| **A/Myanmar/17MP013/2017** | 2017-06-17 | **EPI_ISL_333358** | EPI1332047 | EPI1332048 | EPI1370314 | EPI1370315 | EPI1370316 | EPI1370317 | EPI1370318 | EPI1370319 |
| **A/Myanmar/17MP014/2017** | 2017-06-17 | **EPI_ISL_333404** | EPI1332294 | EPI1332295 | EPI1370320 | EPI1370321 | EPI1370322 | EPI1370323 | EPI1370324 | EPI1370325 |
| **A/Myanmar/17MP015/2017** | 2017-06-17 | **EPI_ISL_333405** | EPI1332296 | EPI1332297 | EPI1371747 | EPI1371748 | EPI1371749 | EPI1371750 | EPI1371746 | EPI1371751 |
| **A/Myanmar/17MP018/2017** | 2017-06-22 | **EPI_ISL_333406** | EPI1332298 | EPI1332299 | EPI1371752 | EPI1371753 | EPI1371754 | EPI1371755 | EPI1371756 | EPI1371757 |
| **A/Myanmar/17MP019/2017** | 2017-06-22 | **EPI_ISL_333407** | EPI1332300 | EPI1332301 | EPI1371758 | EPI1371759 | EPI1371760 | EPI1371761 | EPI1371762 | EPI1371763 |
| **A/Myanmar/17MP021/2017** | 2017-06-22 | **EPI_ISL_333408** | EPI1332302 | EPI1332303 | EPI1371764 | EPI1371765 | EPI1371766 | EPI1371767 | EPI1371768 | EPI1371769 |
| **A/Myanmar/17M007/2017** | 2017-06-12 | **EPI_ISL_333409** | EPI1332304 | EPI1332305 | EPI1371770 | EPI1371771 | EPI1371772 | EPI1371773 | EPI1371774 | EPI1371775 |
| **A/Myanmar/17M012/2017** | 2017-06-17 | **EPI_ISL_333410** | EPI1332336 | EPI1332337 | EPI1371776 | EPI1371777 | EPI1371778 | EPI1371779 | EPI1371780 | EPI1371781 |
| **A/Myanmar/17M015/2017** | 2017-06-17 | **EPI_ISL_333411** | EPI1332338 | EPI1332339 | EPI1371782 | EPI1371783 | EPI1371784 | EPI1371785 | EPI1371786 | EPI1371787 |
| **A/Myanmar/17M023/2017** | 2017-06-22 | **EPI_ISL_333412** | EPI1332340 | EPI1332341 | EPI1371926 | EPI1371927 | EPI1371928 | EPI1371929 | EPI1371930 | EPI1371931 |
| **A/Myanmar/17M025/2017** | 2017-06-22 | **EPI_ISL_333413** | EPI1332342 | EPI1332343 | EPI1371932 | EPI1371933 | EPI1371934 | EPI1371935 | EPI1371936 | EPI1371937 |
| **A/Myanmar/17M062/2017** | 2017-06-26 | **EPI_ISL_333414** | EPI1332344 | EPI1332345 | EPI1371938 | EPI1371939 | EPI1371940 | EPI1371941 | EPI1371942 | EPI1371943 |
| **A/Myanmar/17M064/2017** | 2017-07-1 | **EPI_ISL_333415** | EPI1332346 | EPI1332347 | EPI1371944 | EPI1371945 | EPI1371946 | EPI1371947 | EPI1371948 | EPI1371949 |
| **A/Myanmar/17M083/2017** | 2017-07-3 | **EPI_ISL_333416** | EPI1332348 | EPI1332349 | EPI1371950 | EPI1371951 | EPI1371952 | EPI1371953 | EPI1371954 | EPI1371955 |
| **A/Myanmar/17M108/2017** | 2017-12-07 | **EPI_ISL_333417** | EPI1332350 | EPI1332351 | EPI1371956 | EPI1371957 | EPI1371958 | EPI1371959 | EPI1371960 | EPI1371961 |
| **A/Myanmar/17M109/2017** | 2017-12-17 | **EPI_ISL_333418** | EPI1332352 | EPI1332353 | EPI1371962 | EPI1371963 | EPI1371964 | EPI1371965 | EPI1371966 | EPI1371967 |
| **A/Myanmar/17M115/2017** | 2017-12-07 | **EPI_ISL_333419** | EPI1332354 | EPI1332355 | EPI1371968 | EPI1371969 | EPI1371970 | EPI1371971 | EPI1371972 | EPI1371973 |
| **A/Myanmar/17M204/2017** | 2017-06-30 | **EPI_ISL_333420** | EPI1332356 | EPI1332357 | EPI1371974 | EPI1371975 | EPI1371976 | EPI1373431 | EPI1371977 | EPI1371978 |
| **A/Myanmar/17M307/2017** | 2017-08-07 | **EPI_ISL_333425** | EPI1332366 | EPI1332367 | EPI1629874 | EPI1629873 | EPI1629872 | EPI1629870 | EPI1629869 | EPI1629871 |
