# Supplementary material for: Epidemic of influenza A(H1N1)pdm09 analyzed by full genome sequences and the first case of oseltamivir-resistant strain in Myanmar 2017
Source: PLoS One. 2020 Mar 4;15(3):e0229601. doi: 10.1371/journal.pone.0229601 (PMC7055873; doi:10.1371/journal.pone.0229601)
Supplement: S2 Table — All the data are registered to the Global Initiative on Sharing All Influenza Data (GISAID). (DOCX) [file pone.0229601.s002.docx]

**S2 Table. Details of influenza A(H1N1)pdm09, HA, and NA sequences used in the phylogenetic tree in this study. All the data are registered at the Global Initiative on Sharing All Influenza Data (GISAID).**

| **Strain name** | **Collection date** | **Isolate ID** | **HA** | **NA** |
| --- | --- | --- | --- | --- |
| **A/Myanmar/17M305/2017** | 2017/07/30 | EPI_ISL_333421 | EPI1332358 | EPI1332359 |
| **A/Myanmar/17M306/2017** | 2017/07/31 | EPI_ISL_333422 | EPI1332360 | EPI1332361 |
| **A/Myanmar/17M307/2017** | 2017/08/07 | EPI_ISL_333425 | EPI1332366 | EPI1332367 |
| **A/Myanmar/17M308/2017** | 2017/08/09 | EPI_ISL_333424 | EPI1332364 | EPI1332365 |
| **A/Myanmar/17M309/2017** | 2017/08/09 | EPI_ISL_333423 | EPI1332362 | EPI1332363 |
| **A/Myanmar/17M310/2017** | 2017/08/11 | EPI_ISL_333426 | EPI1332368 | EPI1332369 |
| A/Gunma/17G008/2018 | 2018/01/15 | EPI_ISL_340076 | EPI1364602 | EPI1364601 |
| A/Gunma/17G013/2018 | 2018/01/16 | EPI_ISL_340077 | EPI1364603 | EPI1364604 |
| A/Gunma/17G020/2018 | 2018/01/19 | EPI_ISL_340078 | EPI1364605 | EPI1364606 |
| A/Hokkaido/17H05/2018 | 2017/12/07 | EPI_ISL_340101 | EPI1364659 | EPI1364660 |
| A/Hokkaido/17H026/2017 | 2017/12/21 | EPI_ISL_340102 | EPI1364662 | EPI1364661 |
| A/Kyoto/17K003/2017 | 2017/12/15 | EPI_ISL_340103 | EPI1364663 | EPI1364664 |
| A/Kyoto/17K012/2017 | 2017/12/15 | EPI_ISL_340682 | EPI1366158 | EPI1366158 |
| A/Kyoto/17K023/2018 | 2018/01/20 | EPI_ISL_340683 | EPI1366158 | EPI1366158 |
| A/Nagasaki/17N003/2017 | 2017/12/05 | EPI_ISL_340104 | EPI1364666 | EPI1364665 |
| A/Nagasaki/17N012/2018 | 2018/01/11 | EPI_ISL_340105 | EPI1364667 | EPI1364668 |
| A/Nagasaki/17N029/2018 | 2018/01/15 | EPI_ISL_340106 | EPI1364698 | EPI1364676 |
| A/Nagasaki/17N048/2018 | 2018/01/09 | EPI_ISL_340110 | EPI1364731 | EPI1364732 |
| A/Nagasaki/17N052/2018 | 2018/01/09 | EPI_ISL_340115 | EPI1364734 | EPI1364733 |
| A/Nagasaki/17N068/2018 | 2018/01/18 | EPI_ISL_340116 | EPI1364735 | EPI1364736 |
| A/Nara/17R003/2017 | 2017/12/7 | EPI_ISL_340121 | EPI1364776 | EPI1364775 |
| A/Nara/17R023/2017 | 2017/12/21 | EPI_ISL_340122 | EPI1364777 | EPI1364778 |
| A/Nara/17R044/2018 | 2018_01_16 | EPI_ISL_340123 | EPI1364780 | EPI1364779 |
| A/Nara/17R052/2018 | 2018_01_18 | EPI_ISL_340124 | EPI1364781 | EPI1364782 |
| A/Nara/17R060/2018 | 2018_01_22 | EPI_ISL_340125 | EPI1364784 | EPI1364783 |
| A/Nara/17R073/2018 | 2018_01_29 | EPI_ISL_340126 | EPI1364785 | EPI1364786 |
| A/Shizuoka/17S005/2017 | 2017/12/15 | EPI_ISL_340684 | EPI1366162 | EPI1366163 |
| A/Shizuoka/17S028/2018 | 2018/01/22 | EPI_ISL_340127 | EPI1364788 | EPI1364787 |
| A/Shizuoka/17S059/2018 | 2018/01/24 | EPI_ISL_340131 | EPI1364792 | EPI1364793 |
| A/Shizuoka/17S064/2018 | 2018/01/26 | EPI_ISL_340132 | EPI1364795 | EPI1364794 |
| A/Okinawa/17T010/2017 | 2017/10/17 | EPI_ISL_340133 | EPI1364796 | EPI1364797 |
| A/Okinawa/17T012/2017 | 2017/10/21 | EPI_ISL_340134 | EPI1364798 | EPI1364799 |
| A/Okinawa/17T013/2017 | 2017/10/23 | EPI_ISL_340135 | EPI1364800 | EPI1364801 |
| A/Okinawa/17T014/2017 | 2017/10/23 | EPI_ISL_340136 | EPI1364802 | EPI1364803 |
| A/Okinawa/17T016/2017 | 2017/11/6 | EPI_ISL_340137 | EPI1364804 | EPI1364805 |
| A/Okinawa/17T022/2017 | 2017/11/25 | EPI_ISL_340138 | EPI1364806 | EPI1364829 |
| A/Okinawa/17T025/2017 | 2017/11/28 | EPI_ISL_340144 | EPI1364851 | No |
| A/Okinawa/17T027/2017 | 2017/12/8 | EPI_ISL_340145 | EPI1364852 | No |
| A/Okinawa/17T029/2017 | 2017/12/11 | EPI_ISL_340149 | EPI1364880 | EPI1364881 |
| A/Okinawa/17T030/2017 | 2017/12/12 | EPI_ISL_340173 | EPI1364987 | No |
| A/Okinawa/17T032/2017 | 2017/12/14 | EPI_ISL_340181 | EPI1365751 | No |
| A/Okinawa/17T034/2017 | 2017/12/19 | EPI_ISL_340422 | EPI1365752 | No |
| A/Okinawa/17T035/2017 | 2017/12/19 | EPI_ISL_340423 | EPI1365753 | No |
| A/Okinawa/17T036/2017 | 2017/12/20 | EPI_ISL_340424 | EPI1365754 | EPI1365755 |
| A/Okinawa/17T037/2017 | 2017/12/21 | EPI_ISL_340425 | EPI1365756 | No |
| A/Okinawa/17T040/2018 | 2018/1/4 | EPI_ISL_340426 | EPI1365758 | No |
| A/Okinawa/17T044/2018 | 2018/1/10 | EPI_ISL_340427 | EPI1365759 | EPI1365886 |
| A/Okinawa/17T045/2018 | 2018/1/15 | EPI_ISL_340449 | EPI1365887 | No |
| A/Okinawa/17T058/2018 | 2018/2/6 | EPI_ISL_340450 | EPI1365888 | EPI1365889 |
