# Supplementary material for: Epidemic of influenza A(H1N1)pdm09 analyzed by full genome sequences and the first case of oseltamivir-resistant strain in Myanmar 2017
Source: PLoS One. 2020 Mar 4;15(3):e0229601. doi: 10.1371/journal.pone.0229601 (PMC7055873; doi:10.1371/journal.pone.0229601)
Supplement: S3 Table — (DOCX) [file pone.0229601.s003.docx]

**S3 Table. Details of amino acid substitutions found in PB2, PB1, PA, NP, MP, and NS protein of influenza A(H1N1)pdm09 viruses collected from out-patients and in-patients in Myanmar compared with viruses from India in 2017**

| **Substitution**  **Strain name** | **PB2** |  | | | | | | | | | | | | | | **PB1** | |  | | | | | | | | | | | | | | | | |
| --- | --- | --- | --- | --- | --- | --- | --- | --- | --- | --- | --- | --- | --- | --- | --- | --- | --- | --- | --- | --- | --- | --- | --- | --- | --- | --- | --- | --- | --- | --- | --- | --- | --- | --- |
|  | **66** | **221** | **231** | **296** | **299** | **355** | **368** | **392** | **398** | **453** | **458** | **475** | **559** | **567** | **612** | **12** | **111** | | **175** | | **197** | | **211** | | **375** | | **379** | | **398** | | **661** | | **733** | |
| **A/Michigan/45/2015** | **I** | **A** | **V** | **D** | **R** | **R** | **R** | **Q** | **T** | **P** | **M** | **L** | **I** | **D** | **T** | **I** | **M** | | **N** | | **K** | | **R** | | **S** | | **K** | | **D** | | **A** | | **G** | |
| **In-patients** |  | | | | | | | | | | | | | | | | | | | | | | | | | | | | | | | | | |
| A/Myanmar/17MP001/2017 |  |  |  |  | K |  |  |  | I | T |  |  |  |  |  |  | |  | | D | | R | |  | |  | |  | | G | |  | |  |
| A/Myanmar/17MP002/2017 | T |  |  |  | K |  |  |  | I | T |  |  |  |  |  | V | |  | |  | |  | |  | | N | |  | |  | |  | |  |
| A/Myanmar/17MP003/2017 | T |  |  |  | K |  |  |  | I | T |  |  |  |  |  | V | |  | |  | |  | |  | | N | |  | |  | |  | |  |
| A/Myanmar/17MP004/2017 | T |  |  |  | K |  |  |  | I | T |  |  |  |  |  | V | |  | |  | |  | |  | | N | |  | |  | |  | |  |
| A/Myanmar/17MP005/2017 | T |  |  |  | K |  |  |  | I | T |  |  |  |  |  | V | |  | |  | |  | |  | | N | |  | |  | |  | | E |
| A/Myanmar/17MP009/2017 | T |  |  |  | K |  |  |  | I | T |  |  |  |  |  | V | |  | |  | |  | |  | | N | |  | |  | |  | |  |
| A/Myanmar/17MP013/2017 | T |  |  |  | K |  |  |  | I | T |  |  |  |  | A | V | |  | |  | |  | |  | | N | |  | |  | |  | |  |
| A/Myanmar/17MP014/2017 | T |  |  |  | K |  |  |  | I | T |  |  |  |  |  | V | |  | |  | |  | |  | | N | |  | |  | | S | |  |
| A/Myanmar/17MP015/2017 | T |  |  |  | K |  |  |  | I | T |  |  |  |  |  | V | |  | |  | |  | |  | | N | |  | |  | |  | |  |
| A/Myanmar/17MP018/2017 | T |  |  |  | K |  |  |  | I | T |  |  |  |  |  | V | |  | |  | |  | |  | | N | |  | |  | |  | |  |
| A/Myanmar/17MP019/2017 | T |  |  |  | K |  |  |  | I | T |  |  |  |  |  | V | |  | |  | |  | |  | | N | |  | |  | |  | |  |
| A/Myanmar/17MP021/2017 | T |  |  |  | K |  |  |  | I | T |  |  |  |  |  | V | |  | |  | |  | |  | | N | |  | |  | |  | |  |
| **Out-patients** |  | | | | | | | | | | | | | | | | | | | | | | | | | | | | | | | | | |
| A/Myanmar/17M007/2017 | T |  |  |  | K |  |  |  | I | T |  |  |  |  |  | V | |  | |  | |  | |  | | N | |  | |  | |  | |  |
| A/Myanmar/17M012/2017 | T |  |  |  | K |  |  |  | I | T |  |  |  |  |  | V | |  | |  | |  | |  | | N | |  | |  | |  | |  |
| A/Myanmar/17M015/2017 | T |  |  |  | K |  |  |  | I | T |  |  |  |  |  | V | |  | |  | |  | |  | | N | |  | |  | |  | |  |
| A/Myanmar/17M023/2017 | T |  |  |  | K |  |  |  | I | T |  |  | T |  |  | V | |  | |  | |  | |  | | N | |  | |  | |  | |  |
| A/Myanmar/17M025/2017 | T |  |  |  | K |  |  |  | I | T |  |  |  |  |  | V | |  | |  | |  | |  | | N | |  | |  | |  | |  |
| A/Myanmar/17M062/2017 | T |  |  |  | K |  |  |  | I | T |  |  |  |  |  | V | |  | |  | |  | |  | | N | |  | |  | |  | |  |
| A/Myanmar/17M064/2017 | T |  |  |  | K |  |  |  | I | T |  |  |  |  |  | V | |  | |  | |  | |  | | N | |  | |  | |  | |  |
| A/Myanmar/17M083/2017 | T |  | M |  | K |  |  |  | I | T | L |  |  | E |  | V | |  | |  | |  | |  | | N | |  | |  | |  | |  |
| A/Myanmar/17M108/2017 | T |  |  |  | K |  |  |  | I | T |  | M |  |  |  | V | |  | |  | |  | |  | | N | |  | |  | |  | |  |
| A/Myanmar/17M109/2017 |  |  |  |  | K | G |  |  | I | T |  |  |  |  |  |  | |  | | D | | R | |  | | N | |  | |  | |  | |  |
| A/Myanmar/17M115/2017 | T |  |  |  | K |  |  |  | I | T |  |  |  |  |  | V | |  | |  | |  | |  | | N | |  | |  | |  | |  |
| A/Myanmar/17M204/2017 |  |  |  |  |  |  |  |  | I | T |  |  |  |  |  |  | | I | |  | |  | |  | |  | |  | |  | |  | |  |
| A/Myanmar/17M307/2017 |  |  |  |  | K |  |  |  | I | T |  |  |  |  |  |  | | I | |  | |  | |  | |  | |  | |  | |  | |  |
| **India** |  | | | | | | | | | | | | | | | | | | | | | | | | | | | | | | | | | |
| A/India/0217/2017 |  |  |  |  | K |  |  |  | I | T |  |  |  |  |  |  | |  | |  | |  | |  | |  | |  | |  | |  | |  |
| A/India/0225/2017 |  |  |  |  | K |  |  |  | I | T |  |  |  |  |  |  | |  | |  | |  | |  | |  | |  | |  | |  | |  |
| A/India/0298/2017 |  |  |  |  | K |  |  |  | I | T |  |  |  |  |  |  | |  | |  | |  | |  | |  | |  | |  | |  | |  |
| A/India/0402/2017 |  |  |  |  | K |  | K |  | I | T |  |  |  |  |  |  | |  | |  | |  | |  | |  | |  | |  | |  | |  |
| A/India/1706/2017 |  |  |  |  | K |  |  | X | I | T |  |  |  |  |  |  | |  | |  | |  | |  | |  | |  | |  | |  | |  |
| A/India/7078/2017 |  |  |  |  | K |  |  |  | I | T |  |  |  |  |  |  | |  | |  | |  | |  | |  | | R | |  | |  | |  |
| A/India/8161/2017 |  | S |  | N | K |  |  |  | I | T |  |  |  |  |  |  | |  | |  | |  | | K | |  | |  | |  | |  | |  |
| A/India/9358/2017 |  |  |  |  | K |  |  |  | I | T |  |  |  |  |  |  | |  | |  | |  | |  | |  | |  | |  | |  | |  |

All analyses were performed by FluSurver (<https://flusurver.bii.a-star.edu.sg/>). A/Michigan/45/2015 was used as a reference automatically. Significance level is assigned by FluSurver depending on the known or predicted biological effect of the mutation

**S3 Table. Details of amino acid substitutions found in PB2, PB1, PA, NP, MP, and NS protein of influenza A(H1N1)pdm09 viruses collected from out-patients and in-patients in Myanmar compared with viruses from India in 2017 (continued)**

| **Substitution**  **Strain name** | **PA** | |  | | | | | | | | | | | | | **MP (M2)** |  | | | **MP**  **(M1)** | **NP** |  | | |
| --- | --- | --- | --- | --- | --- | --- | --- | --- | --- | --- | --- | --- | --- | --- | --- | --- | --- | --- | --- | --- | --- | --- | --- | --- |
|  | **20** | | **58** | **140** | **256** | **272** | **319** | **343** | **348** | **361** | **438** | **487** | **530** | **633** | **675** | **14** | **21** | **27^a^** | **61** | **246** | **34** | **129** | **239** | **451** |
| **A/Michigan/45/2015** | **A** | | **G** | **S** | **K** | **D** | **E** | **A** | **I** | **K** | **I** | **S** | **P** | **I** | **N** | **I** | **G** | **V** | **R** | **V** | **G** | **A** | **M** | **A** |
| **In-patients** |  |  | | | | | | | | | | | | | | | | | | | | | | |
| A/Myanmar/17MP001/2017 |  | |  |  |  |  |  |  |  |  | V |  |  |  |  |  |  |  |  |  | A |  |  |  |
| A/Myanmar/17MP002/2017 |  | |  |  |  |  |  |  |  |  |  |  |  |  |  |  |  | A |  |  |  |  |  |  |
| A/Myanmar/17MP003/2017 |  | |  |  |  |  |  |  |  |  |  |  |  |  |  |  |  | A |  |  |  |  |  |  |
| A/Myanmar/17MP004/2017 |  | |  |  |  |  |  |  |  |  |  |  |  |  | D |  |  | A |  |  |  |  |  |  |
| A/Myanmar/17MP005/2017 |  | |  |  |  |  |  |  |  |  |  |  |  |  |  |  |  | A |  |  |  |  |  |  |
| A/Myanmar/17MP009/2017 |  | |  |  |  |  |  |  |  |  |  |  |  |  |  |  |  | A |  |  |  |  |  |  |
| A/Myanmar/17MP013/2017 |  | |  | A |  | V |  |  |  |  |  |  |  |  |  | V |  | A |  |  |  |  |  |  |
| A/Myanmar/17MP014/2017 |  | |  |  |  |  |  |  |  |  |  |  |  |  |  |  |  | A |  |  |  |  |  |  |
| A/Myanmar/17MP015/2017 |  | |  |  |  |  |  |  |  |  | V | N |  |  |  |  |  | A |  |  |  |  |  |  |
| A/Myanmar/17MP018/2017 |  | |  |  |  |  |  |  |  |  |  |  |  |  |  |  |  | A |  | M |  |  |  |  |
| A/Myanmar/17MP019/2017 |  | |  |  |  |  |  |  |  |  |  |  |  |  |  |  |  | A |  |  |  |  |  |  |
| A/Myanmar/17MP021/2017 |  | |  |  |  |  |  |  |  |  |  |  |  |  |  |  |  | A |  |  |  |  |  |  |
| **Out-patients** |  |  | | | | | | | | | | | | | | | | | | | | | | |
| A/Myanmar/17M007/2017 |  | |  |  |  |  |  |  |  |  |  |  |  |  |  |  |  | A |  |  |  |  |  |  |
| A/Myanmar/17M012/2017 |  | | S |  |  |  |  |  |  |  |  |  |  |  |  |  |  | A |  |  |  |  |  |  |
| A/Myanmar/17M015/2017 |  | |  |  |  |  |  |  |  |  |  |  |  |  |  |  |  | A |  |  |  |  |  |  |
| A/Myanmar/17M023/2017 |  | |  |  | R |  |  |  |  |  |  |  |  | L |  |  |  | A |  |  |  |  |  |  |
| A/Myanmar/17M025/2017 |  | |  |  |  |  |  |  |  |  |  |  |  |  |  |  |  | A |  |  |  |  |  |  |
| A/Myanmar/17M062/2017 |  | |  |  |  |  |  |  |  |  |  |  |  |  |  |  |  | A |  |  |  |  |  |  |
| A/Myanmar/17M064/2017 |  | |  |  |  |  |  |  |  |  |  |  |  |  |  |  |  | A | G |  |  |  |  |  |
| A/Myanmar/17M083/2017 |  | |  |  |  |  |  |  |  |  |  |  |  |  |  |  |  | A |  |  |  |  |  |  |
| A/Myanmar/17M108/2017 |  | |  |  |  |  |  |  |  |  |  |  |  |  |  |  |  | A |  |  |  |  |  |  |
| A/Myanmar/17M109/2017 |  | |  |  |  |  |  |  |  |  | V |  |  |  |  |  |  |  |  |  | A |  |  |  |
| A/Myanmar/17M115/2017 |  | |  |  |  |  |  |  |  |  |  |  |  |  |  |  |  | A |  |  |  |  |  |  |
| A/Myanmar/17M204/2017 |  | |  |  |  |  |  |  |  |  |  |  |  |  |  |  |  |  |  |  |  |  |  |  |
| A/Myanmar/17M307/2017 |  | |  |  |  |  |  |  |  |  |  |  |  |  |  |  | V |  |  |  |  |  |  |  |
| **India** |  |  | | | | | | | | | | | | | | | | | | | | | | |
| A/India/0217/2017 |  | |  |  |  |  |  | E |  |  |  |  |  |  |  |  |  |  |  |  |  |  |  |  |
| A/India/0225/2017 |  | |  |  |  |  |  |  |  | R |  |  |  |  |  |  |  |  |  |  |  |  |  |  |
| A/India/0298/2017 |  | |  |  |  |  | G |  |  |  |  |  |  |  |  |  |  |  |  |  |  |  |  |  |
| A/India/0402/2017 |  | |  |  |  |  |  |  | T |  |  |  |  |  |  |  |  |  |  |  |  |  |  |  |
| A/India/1706/2017 |  | |  |  |  |  |  |  |  |  |  |  |  |  |  |  |  |  |  |  |  |  |  |  |
| A/India/7078/2017 |  | |  |  |  |  |  |  |  |  |  |  | S |  |  |  |  |  |  |  |  |  |  |  |
| A/India/8161/2017 |  | |  |  |  |  |  |  |  |  |  |  |  |  |  |  |  |  |  |  |  |  |  | S |
| A/India/9358/2017 | T | |  |  |  |  |  |  |  |  |  |  |  |  |  |  |  |  |  |  |  | T | V |  |

^a^ Reported to be related to drug resistance to amantadine (most significant).

**S3 Table. Details of amino acid substitutions found in PB2, PB1, PA, NP, MP, and NS protein of influenza A(H1N1)pdm09 viruses collected from out-patients and in-patients in Myanmar compared with viruses from India in 2017 (continued)**

| **Substitution**  **Strain name** | **NS (NS1)** | |  | | | | | | | | **NS (NS2)** |  | | |
| --- | --- | --- | --- | --- | --- | --- | --- | --- | --- | --- | --- | --- | --- | --- |
|  | **20** | | **60** | **65** | **87** | **145** | **160** | **164** | **208** | **209** | **34** | **39** | **115** | **501** |
| **A/Michigan/45/2015** | **K** | | **V** | **M** | **S** | **I** | **I** | **P** | **E** | **N** | **R** | **K** | **A** | **M** |
| **In-patients** |  | | | | | | | | | | | | | |
| A/Myanmar/17MP001/2017 |  | |  | V |  |  |  |  |  | D |  | R |  |  |
| A/Myanmar/17MP002/2017 |  | |  | V |  |  |  |  |  |  |  |  |  |  |
| A/Myanmar/17MP003/2017 |  | |  | V |  |  |  |  |  |  |  |  |  |  |
| A/Myanmar/17MP004/2017 |  | |  | V |  |  |  |  |  |  |  |  |  |  |
| A/Myanmar/17MP005/2017 |  | |  | V |  |  |  |  |  |  |  |  |  |  |
| A/Myanmar/17MP009/2017 |  | |  | V |  |  |  |  |  |  |  |  |  |  |
| A/Myanmar/17MP013/2017 |  | |  | V |  |  |  |  |  |  |  |  |  |  |
| A/Myanmar/17MP014/2017 |  | |  | V |  |  |  |  |  |  |  |  |  |  |
| A/Myanmar/17MP015/2017 |  | |  | V |  | R |  |  |  |  |  |  |  |  |
| A/Myanmar/17MP018/2017 |  | |  | V |  |  |  |  |  |  |  |  |  |  |
| A/Myanmar/17MP019/2017 |  | |  | V |  |  |  |  |  |  |  |  |  |  |
| A/Myanmar/17MP021/2017 | M | |  | V |  |  |  |  |  |  |  |  |  |  |
| **Out-patients** |  | | | | | | | | | | | | | |
| A/Myanmar/17M007/2017 |  |  | | V |  |  |  |  |  |  |  |  |  |  |
| A/Myanmar/17M012/2017 |  |  | | V |  |  |  |  |  |  |  |  |  |  |
| A/Myanmar/17M015/2017 |  |  | | V |  |  |  |  |  |  |  |  |  |  |
| A/Myanmar/17M023/2017 |  |  | | V |  |  |  |  |  |  |  |  |  |  |
| A/Myanmar/17M025/2017 |  |  | | V |  |  |  |  |  |  |  |  |  |  |
| A/Myanmar/17M062/2017 |  |  | | V |  |  |  |  |  |  |  |  |  |  |
| A/Myanmar/17M064/2017 |  |  | | V |  |  |  | H |  |  |  |  |  |  |
| A/Myanmar/17M083/2017 |  |  | | V |  |  |  |  |  |  |  |  |  |  |
| A/Myanmar/17M108/2017 |  |  | | V |  |  |  |  |  |  |  |  |  |  |
| A/Myanmar/17M109/2017 |  |  | | V |  |  |  |  |  | D |  | R |  |  |
| A/Myanmar/17M115/2017 |  |  | | V |  |  |  |  |  |  |  |  |  |  |
| A/Myanmar/17M204/2017 |  |  | | V |  |  |  |  |  |  |  |  | V |  |
| A/Myanmar/17M307/2017 |  |  | | V |  |  | L |  |  |  |  |  |  |  |
| **India** |  | | | | | | | | | | | | | |
| A/India/0217/2017 |  | |  | V |  |  |  |  | K |  |  |  |  | I |
| A/India/0225/2017 |  | |  | V |  |  |  |  |  |  |  |  |  |  |
| A/India/0298/2017 |  | |  | V |  |  |  |  |  |  |  |  |  |  |
| A/India/0402/2017 |  | |  | V |  |  |  |  |  |  |  |  |  |  |
| A/India/1706/2017 |  | |  | V | X | V |  |  |  |  |  |  |  |  |
| A/India/7078/2017 |  | |  | V |  |  |  |  |  |  | Q |  |  |  |
| A/India/8161/2017 |  | | X | V |  |  |  |  |  |  |  |  |  |  |
| A/India/9358/2017 |  | |  | V |  |  |  |  |  |  |  |  |  |  |
