# Supplementary material for: Epidemic of influenza A(H1N1)pdm09 analyzed by full genome sequences and the first case of oseltamivir-resistant strain in Myanmar 2017
Source: PLoS One. 2020 Mar 4;15(3):e0229601. doi: 10.1371/journal.pone.0229601 (PMC7055873; doi:10.1371/journal.pone.0229601)
Supplement: S1 File — Management Protocol and Case Report Form. North Okkalapa General Hospital. (PDF) [file pone.0229601.s004.pdf]

INFLUENZA H1N1 (pdm) INFECTION
MANAGEMENT PROTOCOL & CASE RECORD FORM
NORTH OKKALAPA GENERAL HOSPITAL

✔ need to be ticked in appropriate □ boxes.

INCLUSION CRITERIA FOR SUSPECTED INFLUENZA
All patients presenting with fever and respiratory symptoms (cough and/or sore throat) less than 7 days MUST be managed according to this protocol.

INITIAL ASSESSMENT PLACE
All patients with inclusion criteria must be initially assessed and managed at fever clinic (A&E ward) by AS and nurses.

PATIENT'S IDENTITY			
Name/age/sex			
Date & time of arrival to A&E		Date & time of hospital admission	
Hospital R/N	□ OPD	□ Inpatient	
Current staying address			
Numbers of people at current residence			

CLINICAL, RADIOLOGICAL AND LABORATORY TESTS NEED TO BE DONE IN ALL PATIENTS AT A&E (Results need to be fulfilled before leaving A&E)			
Comorbid diseases:			
Blood pressure	Pulse rate	Respiratory rate	SpO2
□RBS (mg%)	□ Hess Test:	□ Pregnancy Test UCG (if suspect):	
□ECG finding:		□ CXR(PA) finding:	
□ RDT for H1N1 (**ONLY** in Cat B(ii) & C patient)		□HIV Test (if suspect):	Days of fever:
**LABORATORY TESTS NEED TO BE DONE IN SELECTED/ ADMITTED PATIENTS AT A&E (Results No Need to be fulfilled before leaving A&E)**			
□ Blood for CP: WBC ____ , Hb ____ , Plt ____			□ ESR:
□ U&E:	□ Creatinine:	□ LFT(optional): ALT___ , AST___ , Bilirubin ___	

CATEGORIZATION OF PATIENTS	
□ Category A	Mild fever PLUS □cough and/or □ sore throat<br>with or without □ body aches, □ headache, □ diarrhea and □ vomiting
□ Category B	□ B (i) **Category A** PLUS □ high grade fever ≥100.4 °F (≥38°C) and □ severe sore throat
	□ B (ii) **Category A** in people with comorbidities like –<br>a. □ Pregnant women<br>b. □ Lung/ □ heart/ □ liver/ □ kidney/ □ neurological diseases/ □blood disorders/ □ diabetes/ □ cancer/ □ HIV-AIDS<br>c. □ On long term steroids or □ those with immunosuppression due to drugs, radiation or HIV, etc.

Validity of this protocol is effective from 1/8/2017 until updated next time or announcement for termination of influenza surveillance period.

□ Category C	**Category A** PLUS any three or more of the following<br>□ Breathlessness<br>□ Chest pain<br>□ Drowsiness<br>□ Fall in blood pressure (<90/60 mmHg)<br>□ Cyanosis<br>□ Tachypnea (RR >30/min)<br>□ Decreased oxygen saturation (SpO2 <90 on air)<br>□ CXR – patchy opacities

H1N1 TESTING AND TREATMENT

Categories	H1N1 Test	Treatments
□ Category A	No testing needed	No admission and Observe at home<br>Symptomatic treatment<br>(Paracetamol, Vitamin C and adequate fluid, etc.)
□ Category B (i)	No testing needed	No admission and Observe at home<br>Treatments as above + PO antibiotics
□ Category B (ii)	Rapid Diagnosis (Antigen)Test (RDT)	If RDT □ positive,<br>Admit to Medical Ward (Isolation Unit) and treatment is guided by comorbidity conditions.<br>If RDT □ negative, Admit to General Medical Ward
□ Category C	RDT and Nasopharyngeal Swab for H1N1 test (send to NHL)	If RDT □ positive, Admit to Medical Ward (Isolation Unit) **OR** □ Refer to Waibargi Specialist Hospital.<br>If RDT □ negative, Admit to General Medical Ward.

**DUTIES AND RECOMMENDATIONS**
Category A patients have to be handled by A&E fever clinic AS and have to inform to Medical ERC Duty PG.
Category B (i) patients have to be handled by Medical ERC duty PG and have to inform to Medical Duty Team Leader SAS.
Category B (ii) and C patients have to be seen by Medical ERC duty PG and Medical Duty Team Leader SAS and have to inform to Medical Oncall Consultants.
**Referral to Waibargi Specialist Hospital** of Category C patients has to be decided by Oncall Medical Consultant.
Prescribing antiviral treatment to Category C patients has to be decided by Oncall Medical Consultant.
Chemoprophylasis for contact person with positive cases is not recommended.
Patients visiting to specialist OPD who met criteria for suspected influenza MUST be referred to A&E fever clinic by OPD nurses.

DOCTORS & NURSES WHO HANDLED PATIENT AT A&E (FULL NAME IN CAPITAL)

AS.	/N.	PG.	SAS.

H1N1 TEST RESULT (For Cat B(ii) and Cat C patients)

□ RDT	□ PCR	□ RDT and PCR

COMPLICATIONS AND OUTCOME (For Cat B(ii) and Cat C patients)

Complications occurred during hospital stay:

Outcome: patient was □ discharged, □ referred, □ expired at Date _ _ / _ _ / _ _ Time _ _ : _ _

COD (if expired):

NOTICE: This record has to be filled for all included patients by respective AS, PG AS and SAS who managed the patient and attached to patient's treatment file during hospital stay and MUST be filed in H1N1 record file of medical ward after patient was discharged.

Validity of this protocol is effective from 1/8/2017 until updated next time or announcement for termination of influenza surveillance period.
