# Supplementary material for: Epidemic of influenza A(H1N1)pdm09 analyzed by full genome sequences and the first case of oseltamivir-resistant strain in Myanmar 2017
Source: PLoS One. 2020 Mar 4;15(3):e0229601. doi: 10.1371/journal.pone.0229601 (PMC7055873; doi:10.1371/journal.pone.0229601)
Supplement: Attachment — Submitted filename: Response to Reviewers.docx [file pone.0229601.s005.docx]

**Response to Academic Editor and Reviewers**

Thank you for the letter and for the reviewers’ comments. We would like to report that our MS was revised as the requirements and comments from the academic editor and reviewers. We respond to each point raised by the academic editor and reviewers.

**Journal Requirements:**

1. When submitting your revision, we need you to address these additional requirements.

 Please ensure that your manuscript meets PLOS ONE's style requirements, including those for file naming. The PLOS ONE style templates can be found at

<http://www.journals.plos.org/plosone/s/file?id=wjVg/PLOSOne_formatting_sample_main_body.pdf> and <http://www.journals.plos.org/plosone/s/file?id=ba62/PLOSOne_formatting_sample_title_authors_affiliations.pdf>

**Response to comment:**

**Thank you for your suggestion. We have checked and corrected the manuscript according to PLOS ONE’s format.**

**We have done 3 major changes in the revised manuscript. One of the authors has changed from Nobuhiro Takemae to Yuko Uchida due to the mistake of a person who conducted the NGS. We elaborated the information for the in-patients and added the clinical characteristics of the in-patients. Additionally, we also added the internal genes sequence of the H275Y mutated virus analyzed by NGS.**

**Academic Editor’s Comments**

1. Line 58, replace "HI" with "Hemagglutination inhibition (HI)"

**Response to comment:**

**Line 56-59, page 3: We changed as “Hemagglutination inhibition (HI)” and modified the sentence.**

2. Line 121, please provide more information regarding in-patients, how many samples? Are they similar to out-patients in characteristics, etc.

**Response to comment:**

**Line 131-132, 136-140, page 7: We provided more information regarding in-patients including sample collection site, patient symptoms with inclusion and exclusion criteria.**

**Line 272-306, page 13-15: We also revised and modified Table 1 and Table 2 providing more information of in-patients.**

**Line 558-563, page 28-29: We reported our study limitations about in-patients in the discussion part.**

3. Line 184:  replace "MUNANA" with "2′-(4-Methylumbelliferyl)-α-D-N-acetylneuraminic acid (MUNANA)".

**Response to comment:**

**Line 204-205, page 10: We replaced "2′-(4-Methylumbelliferyl)-α-D-N-acetylneuraminic acid (MUNANA)" instead of MUNANA.**

4. Line 192, please add (Niigata, Japan) after Niigata University.

**Response to comment:**

**Line 213, page 10: We added “(Niigata, Japan)” after Niigata University.**

**5.** Line 194 -195, suggest removing "using Sanger Method" from the title

**Response to comment:**

**Line 235-238, page 11-12: We deleted the whole paragraph “Hemagglutinin and Neuraminidase gene sequencing using Sanger Method” and incorporated into the paragraph “Phylogenetic analysis” to reflect the suggestion from reviewer 1, comment 6.**

**Reviewer #1:**

**Minor concerns**
1. The introduction is well written, encompasses some aspects of influenza virology relevant to surveillance and focused properly on the work done here.

**Response to comment:**

**Thank you very much for your comment.**

2. In M& M, Neuraminidase assay section can be written better. It is somehow confusing. For instance in three sequential sentences “FOUR KINDS OF NAI” was repeated.

**Response to comment:**

**Line 198-204, page 10: We changed repeated words “FOUR KINDS OF NAI” and made some changes on Neuraminidase assay section to be better and clear.**

3. In line 196 (pdf version), “The HA and NA proteins of selected A(H1N1)pdm09 viruses” should be “The HA and NA genes of selected A(H1N1)pdm09 viruses”.

**Response to comment:**

**The sentence was deleted during revision as per response to reviewer 1, comment 6.**

4. In results, some data presented in table 1 are repeated in the text unnecessarily, also, demographic and baseline clinical characteristics of A(H1N1)pdm09 patients are clearly explained in table 2 and no need for more explanation. Please present the data just one time; table or text.

**Response to comment:**

**Line 272-306, page 13-15: We presented the data one time mainly in Tables (both Table 1 and 2) and removed duplicate data from text under result section in revised manuscript.**

**We modified Table 1 and 2 and provided more information about in-patients as suggested by academic editor and reviewer 2.**

5. In table 6, please refer to appropriate article when mention to play a role in viral oligomerization interfaces and binding of small ligands/ to be related to virulence when combined with V3 and N328K for an avian virus A/quail/Vietnam/36/04 (H5N1) and to be related to drug resistance to amantadine.(line 452 -455 pdf version)

**Response to comment:**

**We deleted the irrelevant sentence and reference (line 434-436 of original version) as suggested by reviewer 2, comment 13.**

**Line 433, page 22: We added the appropriate article related to drug resistance to amantadine.**

**Line 421-435, page 21-22: We summarized the internal gene analysis of influenza A(H1N1)pdm09 viruses and added the result of oseltamivir-resistant Myanmar out-patient strain (A/Myanmar/17M307/2017) in Table 6 of revised manuscript.**

6. Please explain why did you Sanger sequencing while you could obtain the same data from NGS.

**Response to comment:**

**Line 235-238, page 11-12: In previous manuscript, we did not sequence the oseltamivir-resistant case in NGS but in Sanger method. In revised manuscript, we have done NGS to the oseltamivir-resistant case. As a consequence, the paragraph “Hemagglutinin and Neuraminidase gene sequencing using Sanger Method” incorporated in the paragraph “Phylogenetic analysis” in the revised manuscript.**

**Major concern:**

How you can explain HI results? The isolated viruses reacted to antisera raised against vaccine strain even more than the vaccine strain virus!! It should be Vice Versa. Do you think the data presented in this part is reasonable? Please clearly argue about this issue in discussion.

**Response to comment:**

**Line 469-475, page 25: We added the explanation about this in discussion.**

**Reviewer #2:**

**Major points**

1. Title. I would suggest changing the title to something like – influenza full-genome sequencing for surveillance of pathogenic markers and antiviral resistance during the 2017 season in Myanmar.

**Response to comment:**

**Thank you so much for your kind suggestion to change the title. However, we would like to highlight the outbreak of influenza A(H1N1)pdm09 viruses during the 2017 season in Myanmar and focus the first case of antiviral resistance not only full-genome sequencing analysis in our MS. We kindly request you to accept the MS title to remain as before; “Epidemic of influenza A(H1N1)pdm09 analyzed by full genome sequences and a first case of oseltamivir-resistant strain in Myanmar 2017”.**

2. Abstract. There is no mention on the in-patients here

**Response to comment:**

**Lines 52-54, page 3: The information is added in the abstract.**

3. L121. Please specify location of the YKCH Hospital (Myanmar, I guess)

**Response to comment:**

**Line 132, page 7: We specified the location of YKCH hospital in revised text.**

4. Besides, one of the problems with this paper is that in-patients were selected from a different center from that of out-patients. Why only 12? This limitation needs to be acknowledged in the discussion. Why did you not include as in-patients those patients who were finally admitted in the two surveillance hospitals? The comparison of mild/severe cases from different sites needs an explanation.

**Response to comment:**

**Line 382-383, page 20: We inserted the reason for the numbers of the in-patients sequenced.**

**Line 558-563, page 28-29: We added and explained these limitations in discussion.**

5. L164-165. Please provide a table with the sequences for the primers and probes, or an adequate reference for the work describing those.

**Response to comment:**

**Line 189, page 9: We previously described the appropriate reference number 12 in the original version.**

6. L218. Please, specify details on the software: what is type of treatment to the NGS raw data? What is the output? Only consensus files? Coverage, and variant tables?

**Response to comment:**

**Line 222-231, page 11 and line 351-354, page 18: In this time, the variant rate of oseltamivir resistant amino acid substitution at position 275 in NA was provided but the variant table for other positions was not provided.**

**Line 566-570, page 29: We also added these limitations in discussion of revised manuscript.**

7. L231. The authors used MEGA for the phylogenetic analysis but, what was the evolutionary model best fitting the data (for every segment and for the whole genome)? Dis you consider that in the phylogenetic analysis?

**Response to comment:**

**Line 247-251, page 12: We clarified the best fitting model in the revised manuscript.**

8. L282. For the patients included, did you collect the previous vaccination status?

**Response to comment:**

**Line 297-298, page 14: We collected the previous influenza vaccination status and added it in revised text.**

9. L415 segments, no proteins

**Response to comment:**

**Line 414, page 21: We changed as segments as you suggested.**

10. L420. For the NGS whole-genome sequence there are no details on the results obtained (i.e. mean coverage per segment, depth per nucleotide, variants per segment (synonymous, non-synonymous, major and minor) etc.

**Response to comment:**

**Line 566-570, page 29: The further analysis of the six internal genes and details of the NGS whole-genome sequencing results obtained will be reported in the next paper.**

11. Besides, the analyses performed on NGS data seem limited to the consensus sequences. The authors do not provide a (major and minor) variant analysis for the 8 genome segments, table S3 seems just a summary of alignments using consensus sequences. It would be interesting to see if minor variants were more/less abundant in patients with severe outcome.

**Response to comment:**

**Line 566-570, page 29, line 351-354, page 18, Table 6 and S3 Table: In the revised manuscript, we did not describe the minor variants analysis because much detail analysis on epidemiological findings was needed. In addition, we added the variant rate of oseltamivir-resistance tyrosine amino acid substitution at position 275 in NA gene of one oseltamivir- resistant Myanmar strain in “Drug resistant influenza A(H1N1)pdm09 virus” under results section. We added the internal genes of oseltamivir-resistant H275Y mutated virus.**

12. Further, there is no phylogenetic analysis on the other segments different from HA or NA…nor using the complete genome, data that may be also useful to look for reassortment/recombination.

**Response to comment:**

**Line 566-570, page 29: We added the limitations in discussion as per response to comment 10 and 11.**

13. L434-436. I believe this last sentence may be not relevant for the reader.

**Response to comment:**

**We deleted the irrelevant sentence as your comment.**

14. L533. There were in fact some differences between strains from Myanmar and those from India, see table 6!

**Response to comment:**

**Line 430-435, page 22, line 521-527, page 27: We explained and inserted the additional information and also added the related reference number 25.**

15. L559-577 these two paragraphs show information that can be parly included in the introduction and that can be significantly streamlined, avoiding some details that may not be interesting to the reader.

**Response to comment:**

**Line 96-103, page 5, line 116-119, page 6: We moved some information from here to introduction section and deleted some details.**

16. L581. Here the authors should acknowledge the limitations on patient inclusion raised on the points above.

**Response to comment:**

**Line 558-563, page 28-29: We added the limitations on patient inclusion.**

17. L585. It would be interesting to see if you found any minor variants in NA at positions associated with NAI resistence, discuss here.

**Response to comment:**

**Line 351-354, page 18: We added the result as per response.**

18. L591-593. This can be streamlined to something like -2017 season but failed to identify features in the viral genome responsible for the differential severity of influenza between in- and out-patients…

**Response to comment:**

**Line 576-578, page 29: We were unable to identify viral genome features to differentiate the severity and described it in conclusion section of revised manuscript.**

**Minor points**

19. L236. A/California/07/2009 (H1N1pdm09). Please specify genebank accession number.

**Response to comment:**

**Line 254, page 12: We specified GISAID** **accession number**.

20. L242. Change “alterations” for “implications”

**Response to comment:**

**Line 260, page 12: We changed “alterations” for “implications” as you suggested.**

21. L346. …resistance

**Response to comment:**

**Line 346, page 17: We changed as resistance.**

22. L352 …region did not show the…

**Response to comment:**

**Line 354, page 18: We changed it according to your suggestion.**

23. L388 T314I in HA?

**Response to comment:**

**Line 387, page 20: We described it in revised manuscript.**

24. L408. …was found in one ….

**Response to comment:**

**Line 407, page 21: We changed as you suggested.**

25. L411 …the D451G…

**Response to comment:**

**Line 410, page 21: We added article “the” D451G.**

26. L423 …substitutions (as compared to A/Michigan…)

**Response to comment:**

**Line 422, page 21: We added as you suggested and summarized the internal gene analysis of influenza A(H1N1)pdm09 viruses in revised manuscript.**

27. L426…which MAY play a role…

**Response to comment:**

**The sentence was deleted during revision as we summarized the internal gene analysis in revised manuscript.**
