# Supplementary material for: Epidemic of influenza A(H1N1)pdm09 analyzed by full genome sequences and the first case of oseltamivir-resistant strain in Myanmar 2017
Source: PLoS One. 2020 Mar 4;15(3):e0229601. doi: 10.1371/journal.pone.0229601 (PMC7055873; doi:10.1371/journal.pone.0229601)
Supplement: Attachment — Submitted filename: Response to Reviewers.doc [file pone.0229601.s006.doc]

**Response to Academic Editor and Reviewers**

Thank you for the letter and for the reviewer’s comment. We would like to report that our MS was revised to meet PLOS ONE’s publication criteria. We respond to each point raised by the academic editor and reviewers during the review process. To comply with your suggestions, we requested a professional English language editing company, Editage, to copyedit thoroughly our manuscript for language usage, clarification on technical expression, spelling, and grammar and any typographical or grammatical errors are corrected at revision to help the readability of the paper.

**Reviewer #2:**

1. In point 10 of the answer to reviewer comments, you argue that further analysis and details of the NGS genome sequencing results will be reported "in the next paper"...and added that comment in the text (P29, L566-570). Please change that for a more adequate statement, such as "analysis of NGS data is still ongoing and the results will be published elsewhere"

**Response to comment:**

**Lines 567-568, page 29: We changed the sentence to be a more adequate statement as you suggested.**
