# Supplementary material for: Epidemic of influenza A(H1N1)pdm09 analyzed by full genome sequences and the first case of oseltamivir-resistant strain in Myanmar 2017
Source: PLoS One. 2020 Mar 4;15(3):e0229601. doi: 10.1371/journal.pone.0229601 (PMC7055873; doi:10.1371/journal.pone.0229601)
Supplement: Peer review history

# Peer review: Decision Letter 0

**DOI:** 10.1371/journal.pone.0229601.r001

**Authors:** Baochuan Lin

22 Oct 2019

PONE-D-19-24652

Epidemic of influenza A(H1N1)pdm09 analyzed by full genome sequences and a first case of oseltamivir-resistant strain in Myanmar 2017

PLOS ONE

Dear Dr. Kyaw Win,

Thank you for submitting your manuscript to PLOS ONE. After careful consideration, we feel that it has merit but does not fully meet PLOS ONE’s publication criteria as it currently stands. Therefore, we invite you to submit a revised version of the manuscript that addresses the points raised during the review process.

I have received the reviews of your manuscript. While your paper addresses an interesting question, there are several issues raised by the reviewers that needs to be addressed.  Please see reviewers' insightful comments for detail.  On a personal level, I felt the paper is very descriptive and I agreed with the reviewers about the information for in-patients did not appear until "drug resistance testing", please provide more information regarding in-patients.  Also, some details on the phylogenetic analysis on the whole genome will help strengthen the merit of the paper.

Specific comments:

1. Line 58, replace "HI" with "Hemagglutination inhibition (HI)"

2. Line 121, please provide more information regarding in-patients, how many samples? Are they similar to out-patients in characteristics, etc.

3. Line 184:  replace "MUNANA" with "2′-(4-Methylumbelliferyl)-α-D-N-acetylneuraminic acid (MUNANA)".

4. Line 192, please add (Niigata, Japan) after Niigata University.

5. Line 194 - 195, suggest removing "using Sanger Method" from the title

We would appreciate receiving your revised manuscript by Dec 06 2019 11:59PM. When you are ready to submit your revision, log on to https://www.editorialmanager.com/pone/ and select the 'Submissions Needing Revision' folder to locate your manuscript file.

If you would like to make changes to your financial disclosure, please include your updated statement in your cover letter.

To enhance the reproducibility of your results, we recommend that if applicable you deposit your laboratory protocols in protocols.io, where a protocol can be assigned its own identifier (DOI) such that it can be cited independently in the future. For instructions see: http://journals.plos.org/plosone/s/submission-guidelines#loc-laboratory-protocols

Please include the following items when submitting your revised manuscript:

- A rebuttal letter that responds to each point raised by the academic editor and reviewer(s). This letter should be uploaded as separate file and labeled 'Response to Reviewers'.
- A marked-up copy of your manuscript that highlights changes made to the original version. This file should be uploaded as separate file and labeled 'Revised Manuscript with Track Changes'.
- An unmarked version of your revised paper without tracked changes. This file should be uploaded as separate file and labeled 'Manuscript'.

Please note while forming your response, if your article is accepted, you may have the opportunity to make the peer review history publicly available. The record will include editor decision letters (with reviews) and your responses to reviewer comments. If eligible, we will contact you to opt in or out.

We look forward to receiving your revised manuscript.

Kind regards,

Baochuan Lin, Ph.D.

Academic Editor

PLOS ONE

Journal Requirements:

1.

When submitting your revision, we need you to address these additional requirements.

Please ensure that your manuscript meets PLOS ONE's style requirements, including those for file naming. The PLOS ONE style templates can be found at

http://www.journals.plos.org/plosone/s/file?id=wjVg/PLOSOne_formatting_sample_main_body.pdf and http://www.journals.plos.org/plosone/s/file?id=ba62/PLOSOne_formatting_sample_title_authors_affiliations.pdf

[Note: HTML markup is below. Please do not edit.]

Reviewers' comments:

Reviewer's Responses to Questions

**Comments to the Author**

1. Is the manuscript technically sound, and do the data support the conclusions?

The manuscript must describe a technically sound piece of scientific research with data that supports the conclusions. Experiments must have been conducted rigorously, with appropriate controls, replication, and sample sizes. The conclusions must be drawn appropriately based on the data presented. 

Reviewer #1: Yes

Reviewer #2: Partly

**********

2. Has the statistical analysis been performed appropriately and rigorously? 

Reviewer #1: Yes

Reviewer #2: N/A

**********

3. Have the authors made all data underlying the findings in their manuscript fully available?

The PLOS Data policy requires authors to make all data underlying the findings described in their manuscript fully available without restriction, with rare exception (please refer to the Data Availability Statement in the manuscript PDF file). The data should be provided as part of the manuscript or its supporting information, or deposited to a public repository. For example, in addition to summary statistics, the data points behind means, medians and variance measures should be available. If there are restrictions on publicly sharing data—e.g. participant privacy or use of data from a third party—those must be specified.

Reviewer #1: Yes

Reviewer #2: No

**********

4. Is the manuscript presented in an intelligible fashion and written in standard English?

PLOS ONE does not copyedit accepted manuscripts, so the language in submitted articles must be clear, correct, and unambiguous. Any typographical or grammatical errors should be corrected at revision, so please note any specific errors here.

Reviewer #1: Yes

Reviewer #2: No

**********

5. Review Comments to the Author

Please use the space provided to explain your answers to the questions above. You may also include additional comments for the author, including concerns about dual publication, research ethics, or publication ethics. (Please upload your review as an attachment if it exceeds 20,000 characters)

Reviewer #1: This is a descriptive study on H1N1 influenza viruses isolated from in-patients and out-patients (mostly less than 14 years old) in Myanmar in 2017. It was motivated following a community outbreak during this year and declaration a high level of alert.

The authors investigated the circulation patterns, distribution of influenza subtypes, antigenic and genetic characterization of influenza A(H1N1)pdm09 and susceptibility to neuraminidase inhibitors.

The virus circulating in Myanmar was found to have several HA mutations including S84N, S162N and I216T; and NA mutations including V13I, I34V and I314M substitutions which placed the circulating H1N1 virus in the 6.1B clade.

Minor concerns

1. The introduction is well written, encompasses some aspects of influenza virology relevant to surveillance and focused properly on the work done here.

2. In M& M, Neuraminidase assay section can be written better. It is somehow confusing. For instance in three sequential sentences “FOUR KINDS OF NAI” was repeated.

3. In line 196 (pdf version), “The HA and NA proteins of selected A(H1N1)pdm09 viruses” should be “The HA and NA genes of selected A(H1N1)pdm09 viruses”.

4. In results, some data presented in table 1 are repeated in the text unnecessarily, also, demographic and baseline clinical characteristics of A(H1N1)pdm09 patients are clearly explained in table 2 and no need for more explanation. Please present the data just one time; table or text.

5. In table 6, please refer to appropriate article when mention to play a role in viral oligomerization interfaces and binding of small ligands/ to be related to virulence when combined with V3 and N328K for an avian virus A/quail/Vietnam/36/04 (H5N1) and to be related to drug resistance to amantadine.(line 452 -455 pdf version)

6. Please explain why did you Sanger sequencing while you could obtain the same data from NGS.

Major concern:

How you can explain HI results? The isolated viruses reacted to antisera raised against vaccine strain even more than the vaccine strain virus!! It should be Vice Versa. Do you think the data presented in this part is reasonable? Please clearly argue about this issue in discussion.

Reviewer #2: MAJOR POINTS

1. Title. I would suggest changing the title to something like – influenza full-genome sequencing for surveillance of pathogenic markers and antiviral resistance during the 2017 season in Myanmar.

2. Abstract. There is no mention on the in-patients here

3. L121. Please specify location of the YKCH Hospital (Myanmar, I guess)

4. Besides, one of the problems with this paper is that in-patients were selected from a different center from that of out-patients. Why only 12? This limitation needs to be acknowledged in the discussion. Why did you not include as in-patients those patients who were finally admitted in the two surveillance hospitals? The comparison of mild/severe cases from different sites needs an explanation.

5. L164-165. Please provide a table with the sequences for the primers and probes, or an adequate reference for the work describing those.

6. L218. Please, specify details on the software: what is type of treatment to the NGS raw data? What is the output? Only consensus files? Coverage, and variant tables?

7. L231. The authors used MEGA for the phylogenetic analysis but, what was the evolutionary model best fitting the data (for every segment and for the whole genome)? Dis you consider that in the phylogenetic analysis?

8. L282. For the patients included, did you collect the previous vaccination status?

9. L415 segments, no proteins

10. L420. For the NGS whole-genome sequence there are no details on the results obtained (i.e. mean coverage per segment, depth per nucleotide, variants per segment (synonymous, non-synonymous, major and minor) etc.

11. Besides, the analyses performed on NGS data seem limited to the consensus sequences. The authors do not provide a (major and minor) variant analysis for the 8 genome segments, table S3 seems just a summary of alignments using consensus sequences. It would be interesting to see if minor variants were more/less abundant in patients with severe outcome.

12. Further, there is no phylogenetic analysis on the other segments different from HA or NA…nor using the complete genome, data that may be also useful to look for reassortment/recombination.

13. L434-436. I believe this last sentence may be not relevant for the reader.

14. L533. There were in fact some differences between strains from Myanmar and those from India, see table 6!

15. L559-577 these two paragraphs show information that can be parly included in the introduction and that can be significantly streamlined, avoiding some details that may not be interesting to the reader.

16. L581. Here the authors should acknowledge the limitations on patient inclusion raised on the points above.

17. L585. It would be interesting to see if you found any minor variants in NA at positions associated with NAI resistence, discuss here.

18. L591-593. This can be streamlined to something like -2017 season but failed to identify features in the viral genome responsible for the differential severity of influenza between in- and out-patients…

MINOR POINTS

19. L236. A/California/07/2009 (H1N1pdm09). Please specify genebank accession number.

20. L242. Change “alterations” for “implications”

21. L346. …resistance

22. L352 …region did not show the…

23. L388 T314I in HA?

24. L408. …was found in one ….

25. L411 …the D451G…

26. L423 …substitutions (as compared to A/Michigan…)

27. L426…which MAY play a role…

**********

6. PLOS authors have the option to publish the peer review history of their article (what does this mean?). If published, this will include your full peer review and any attached files.

If you choose “no”, your identity will remain anonymous but your review may still be made public.

**Do you want your identity to be public for this peer review?** For information about this choice, including consent withdrawal, please see our Privacy Policy.

Reviewer #1: Yes: first name: Fatemeh, last name: Fotouhi

Reviewer #2: No

[NOTE: If reviewer comments were submitted as an attachment file, they will be attached to this email and accessible via the submission site. Please log into your account, locate the manuscript record, and check for the action link "View Attachments". If this link does not appear, there are no attachment files to be viewed.]

While revising your submission, please upload your figure files to the Preflight Analysis and Conversion Engine (PACE) digital diagnostic tool, https://pacev2.apexcovantage.com/. PACE helps ensure that figures meet PLOS requirements. To use PACE, you must first register as a user. Registration is free. Then, login and navigate to the UPLOAD tab, where you will find detailed instructions on how to use the tool. If you encounter any issues or have any questions when using PACE, please email us at figures@plos.org. Please note that Supporting Information files do not need this step.

# Author comment: Author response to Decision Letter 0

**DOI:** 10.1371/journal.pone.0229601.r002

6 Dec 2019

Response to Academic Editor and Reviewers

Thank you for the letter and for the reviewers’ comments. We would like to report that our MS was revised as the requirements and comments from the academic editor and reviewers. We respond to each point raised by the academic editor and reviewers.

Journal Requirements:

1. When submitting your revision, we need you to address these additional requirements.

 Please ensure that your manuscript meets PLOS ONE's style requirements, including those for file naming. The PLOS ONE style templates can be found at

http://www.journals.plos.org/plosone/s/file?id=wjVg/PLOSOne_formatting_sample_main_body.pdf and http://www.journals.plos.org/plosone/s/file?id=ba62/PLOSOne_formatting_sample_title_authors_affiliations.pdf

Response to comment:

Thank you for your suggestion. We have checked and corrected the manuscript according to PLOS ONE’s format.

We have done 3 major changes in the revised manuscript. One of the authors has changed from Nobuhiro Takemae to Yuko Uchida due to the mistake of a person who conducted the NGS. We elaborated the information for the in-patients and added the clinical characteristics of the in-patients. Additionally, we also added the internal genes sequence of the H275Y mutated virus analyzed by NGS. 

Academic Editor’s Comments 

1. Line 58, replace "HI" with "Hemagglutination inhibition (HI)"

Response to comment:

Line 56-59, page 3: We changed as “Hemagglutination inhibition (HI)” and modified the sentence. 

2. Line 121, please provide more information regarding in-patients, how many samples? Are they similar to out-patients in characteristics, etc.

Response to comment:

Line 131-132, 136-140, page 7: We provided more information regarding in-patients including sample collection site, patient symptoms with inclusion and exclusion criteria.

Line 272-306, page 13-15: We also revised and modified Table 1 and Table 2 providing more information of in-patients. 

Line 558-563, page 28-29: We reported our study limitations about in-patients in the discussion part. 

3. Line 184: replace "MUNANA" with "2′-(4-Methylumbelliferyl)-α-D-N-acetylneuraminic acid (MUNANA)".

Response to comment:

Line 204-205, page 10: We replaced "2′-(4-Methylumbelliferyl)-α-D-N-acetylneuraminic acid (MUNANA)" instead of MUNANA.

4. Line 192, please add (Niigata, Japan) after Niigata University.

Response to comment:

Line 213, page 10: We added “(Niigata, Japan)” after Niigata University.

5. Line 194 -195, suggest removing "using Sanger Method" from the title

Response to comment:

Line 235-238, page 11-12: We deleted the whole paragraph “Hemagglutinin and Neuraminidase gene sequencing using Sanger Method” and incorporated into the paragraph “Phylogenetic analysis” to reflect the suggestion from reviewer 1, comment 6.

Reviewer #1:

Minor concerns

1. The introduction is well written, encompasses some aspects of influenza virology relevant to surveillance and focused properly on the work done here.

Response to comment:

Thank you very much for your comment. 

2. In M& M, Neuraminidase assay section can be written better. It is somehow confusing. For instance in three sequential sentences “FOUR KINDS OF NAI” was repeated.

Response to comment:

Line 198-204, page 10: We changed repeated words “FOUR KINDS OF NAI” and made some changes on Neuraminidase assay section to be better and clear.

3. In line 196 (pdf version), “The HA and NA proteins of selected A(H1N1)pdm09 viruses” should be “The HA and NA genes of selected A(H1N1)pdm09 viruses”.

Response to comment:

The sentence was deleted during revision as per response to reviewer 1, comment 6.

4. In results, some data presented in table 1 are repeated in the text unnecessarily, also, demographic and baseline clinical characteristics of A(H1N1)pdm09 patients are clearly explained in table 2 and no need for more explanation. Please present the data just one time; table or text.

Response to comment:

Line 272-306, page 13-15: We presented the data one time mainly in Tables (both Table 1 and 2) and removed duplicate data from text under result section in revised manuscript. 

We modified Table 1 and 2 and provided more information about in-patients as suggested by academic editor and reviewer 2. 

5. In table 6, please refer to appropriate article when mention to play a role in viral oligomerization interfaces and binding of small ligands/ to be related to virulence when combined with V3 and N328K for an avian virus A/quail/Vietnam/36/04 (H5N1) and to be related to drug resistance to amantadine.(line 452 -455 pdf version)

Response to comment:

We deleted the irrelevant sentence and reference (line 434-436 of original version) as suggested by reviewer 2, comment 13. 

Line 433, page 22: We added the appropriate article related to drug resistance to amantadine. 

Line 421-435, page 21-22: We summarized the internal gene analysis of influenza A(H1N1)pdm09 viruses and added the result of oseltamivir-resistant Myanmar out-patient strain (A/Myanmar/17M307/2017) in Table 6 of revised manuscript.

 6. Please explain why did you Sanger sequencing while you could obtain the same data from NGS.

Response to comment:

Line 235-238, page 11-12: In previous manuscript, we did not sequence the oseltamivir-resistant case in NGS but in Sanger method. In revised manuscript, we have done NGS to the oseltamivir-resistant case. As a consequence, the paragraph “Hemagglutinin and Neuraminidase gene sequencing using Sanger Method” incorporated in the paragraph “Phylogenetic analysis” in the revised manuscript.

Major concern:

 How you can explain HI results? The isolated viruses reacted to antisera raised against vaccine strain even more than the vaccine strain virus!! It should be Vice Versa. Do you think the data presented in this part is reasonable? Please clearly argue about this issue in discussion.

Response to comment:

Line 469-475, page 25: We added the explanation about this in discussion. 

Reviewer #2: 

Major points

1. Title. I would suggest changing the title to something like – influenza full-genome sequencing for surveillance of pathogenic markers and antiviral resistance during the 2017 season in Myanmar.

Response to comment:

Thank you so much for your kind suggestion to change the title. However, we would like to highlight the outbreak of influenza A(H1N1)pdm09 viruses during the 2017 season in Myanmar and focus the first case of antiviral resistance not only full-genome sequencing analysis in our MS. We kindly request you to accept the MS title to remain as before; “Epidemic of influenza A(H1N1)pdm09 analyzed by full genome sequences and a first case of oseltamivir-resistant strain in Myanmar 2017”.

2. Abstract. There is no mention on the in-patients here

Response to comment:

Lines 52-54, page 3: The information is added in the abstract.

3. L121. Please specify location of the YKCH Hospital (Myanmar, I guess)

Response to comment:

Line 132, page 7: We specified the location of YKCH hospital in revised text.

4. Besides, one of the problems with this paper is that in-patients were selected from a different center from that of out-patients. Why only 12? This limitation needs to be acknowledged in the discussion. Why did you not include as in-patients those patients who were finally admitted in the two surveillance hospitals? The comparison of mild/severe cases from different sites needs an explanation.

Response to comment:

Line 382-383, page 20: We inserted the reason for the numbers of the in-patients sequenced.

Line 558-563, page 28-29: We added and explained these limitations in discussion. 

5. L164-165. Please provide a table with the sequences for the primers and probes, or an adequate reference for the work describing those.

Response to comment:

Line 189, page 9: We previously described the appropriate reference number 12 in the original version.

6. L218. Please, specify details on the software: what is type of treatment to the NGS raw data? What is the output? Only consensus files? Coverage, and variant tables?

Response to comment:

Line 222-231, page 11 and line 351-354, page 18: In this time, the variant rate of oseltamivir resistant amino acid substitution at position 275 in NA was provided but the variant table for other positions was not provided. 

Line 566-570, page 29: We also added these limitations in discussion of revised manuscript.

7. L231. The authors used MEGA for the phylogenetic analysis but, what was the evolutionary model best fitting the data (for every segment and for the whole genome)? Dis you consider that in the phylogenetic analysis?

Response to comment:

Line 247-251, page 12: We clarified the best fitting model in the revised manuscript. 

8. L282. For the patients included, did you collect the previous vaccination status?

Response to comment:

Line 297-298, page 14: We collected the previous influenza vaccination status and added it in revised text. 

9. L415 segments, no proteins

Response to comment:

Line 414, page 21: We changed as segments as you suggested.

10. L420. For the NGS whole-genome sequence there are no details on the results obtained (i.e. mean coverage per segment, depth per nucleotide, variants per segment (synonymous, non-synonymous, major and minor) etc.

Response to comment:

Line 566-570, page 29: The further analysis of the six internal genes and details of the NGS whole-genome sequencing results obtained will be reported in the next paper. 

11. Besides, the analyses performed on NGS data seem limited to the consensus sequences. The authors do not provide a (major and minor) variant analysis for the 8 genome segments, table S3 seems just a summary of alignments using consensus sequences. It would be interesting to see if minor variants were more/less abundant in patients with severe outcome.

Response to comment:

Line 566-570, page 29, line 351-354, page 18, Table 6 and S3 Table: In the revised manuscript, we did not describe the minor variants analysis because much detail analysis on epidemiological findings was needed. In addition, we added the variant rate of oseltamivir-resistance tyrosine amino acid substitution at position 275 in NA gene of one oseltamivir- resistant Myanmar strain in “Drug resistant influenza A(H1N1)pdm09 virus” under results section. We added the internal genes of oseltamivir-resistant H275Y mutated virus. 

12. Further, there is no phylogenetic analysis on the other segments different from HA or NA…nor using the complete genome, data that may be also useful to look for reassortment/recombination.

Response to comment:

Line 566-570, page 29: We added the limitations in discussion as per response to comment 10 and 11.

13. L434-436. I believe this last sentence may be not relevant for the reader.

Response to comment:

We deleted the irrelevant sentence as your comment.

14. L533. There were in fact some differences between strains from Myanmar and those from India, see table 6!

Response to comment:

Line 430-435, page 22, line 521-527, page 27: We explained and inserted the additional information and also added the related reference number 25.

15. L559-577 these two paragraphs show information that can be parly included in the introduction and that can be significantly streamlined, avoiding some details that may not be interesting to the reader.

Response to comment:

Line 96-103, page 5, line 116-119, page 6: We moved some information from here to introduction section and deleted some details.

16. L581. Here the authors should acknowledge the limitations on patient inclusion raised on the points above.

Response to comment:

Line 558-563, page 28-29: We added the limitations on patient inclusion.

17. L585. It would be interesting to see if you found any minor variants in NA at positions associated with NAI resistence, discuss here.

Response to comment:

Line 351-354, page 18: We added the result as per response. 

18. L591-593. This can be streamlined to something like -2017 season but failed to identify features in the viral genome responsible for the differential severity of influenza between in- and out-patients…

Response to comment:

Line 576-578, page 29: We were unable to identify viral genome features to differentiate the severity and described it in conclusion section of revised manuscript.

Minor points

19. L236. A/California/07/2009 (H1N1pdm09). Please specify genebank accession number.

Response to comment:

Line 254, page 12: We specified GISAID accession number. 

20. L242. Change “alterations” for “implications”

Response to comment:

Line 260, page 12: We changed “alterations” for “implications” as you suggested. 

21. L346. …resistance

Response to comment:

Line 346, page 17: We changed as resistance.

22. L352 …region did not show the…

Response to comment:

Line 354, page 18: We changed it according to your suggestion. 

23. L388 T314I in HA?

Response to comment:

 Line 387, page 20: We described it in revised manuscript.

24. L408. …was found in one ….

Response to comment:

Line 407, page 21: We changed as you suggested. 

25. L411 …the D451G…

Response to comment:

Line 410, page 21: We added article “the” D451G. 

26. L423 …substitutions (as compared to A/Michigan…)

Response to comment:

Line 422, page 21: We added as you suggested and summarized the internal gene analysis of influenza A(H1N1)pdm09 viruses in revised manuscript. 

27. L426…which MAY play a role…

Response to comment:

The sentence was deleted during revision as we summarized the internal gene analysis in revised manuscript.

Attachment Submitted filename: Response to Reviewers.docx Click here for additional data file.

# Peer review: Decision Letter 1

**DOI:** 10.1371/journal.pone.0229601.r003

**Authors:** Baochuan Lin

7 Jan 2020

PONE-D-19-24652R1

Epidemic of influenza A(H1N1)pdm09 analyzed by full genome sequences and a first case of oseltamivir-resistant strain in Myanmar 2017

PLOS ONE

Dear Dr. Kyaw Win,

Thank you for submitting your manuscript to PLOS ONE. After careful consideration, we feel that it has merit but does not fully meet PLOS ONE’s publication criteria as it currently stands. Therefore, we invite you to submit a revised version of the manuscript that addresses the points raised during the review process.

Both reviewers agreed that the revised manuscript showed significant improvement, however, there are some issues that need to be addressed.  Also, please have a fluent, preferably native, English-language speaker thoroughly copyedit your manuscript for language usage, spelling, and grammar since PLoS ONE does not perform copyediting of manuscripts at any later stage in the publication process.

We would appreciate receiving your revised manuscript by Feb 21 2020 11:59PM. When you are ready to submit your revision, log on to https://www.editorialmanager.com/pone/ and select the 'Submissions Needing Revision' folder to locate your manuscript file.

If you would like to make changes to your financial disclosure, please include your updated statement in your cover letter.

To enhance the reproducibility of your results, we recommend that if applicable you deposit your laboratory protocols in protocols.io, where a protocol can be assigned its own identifier (DOI) such that it can be cited independently in the future. For instructions see: http://journals.plos.org/plosone/s/submission-guidelines#loc-laboratory-protocols

Please include the following items when submitting your revised manuscript:

- A rebuttal letter that responds to each point raised by the academic editor and reviewer(s). This letter should be uploaded as separate file and labeled 'Response to Reviewers'.
- A marked-up copy of your manuscript that highlights changes made to the original version. This file should be uploaded as separate file and labeled 'Revised Manuscript with Track Changes'.
- An unmarked version of your revised paper without tracked changes. This file should be uploaded as separate file and labeled 'Manuscript'.

Please note while forming your response, if your article is accepted, you may have the opportunity to make the peer review history publicly available. The record will include editor decision letters (with reviews) and your responses to reviewer comments. If eligible, we will contact you to opt in or out.

We look forward to receiving your revised manuscript.

Kind regards,

Baochuan Lin, Ph.D.

Academic Editor

PLOS ONE

[Note: HTML markup is below. Please do not edit.]

Reviewers' comments:

Reviewer's Responses to Questions

**Comments to the Author**

1. If the authors have adequately addressed your comments raised in a previous round of review and you feel that this manuscript is now acceptable for publication, you may indicate that here to bypass the “Comments to the Author” section, enter your conflict of interest statement in the “Confidential to Editor” section, and submit your "Accept" recommendation.

Reviewer #1: All comments have been addressed

Reviewer #2: All comments have been addressed

**********

2. Is the manuscript technically sound, and do the data support the conclusions?

The manuscript must describe a technically sound piece of scientific research with data that supports the conclusions. Experiments must have been conducted rigorously, with appropriate controls, replication, and sample sizes. The conclusions must be drawn appropriately based on the data presented. 

Reviewer #1: Yes

Reviewer #2: Yes

**********

3. Has the statistical analysis been performed appropriately and rigorously? 

Reviewer #1: Yes

Reviewer #2: Yes

**********

4. Have the authors made all data underlying the findings in their manuscript fully available?

The PLOS Data policy requires authors to make all data underlying the findings described in their manuscript fully available without restriction, with rare exception (please refer to the Data Availability Statement in the manuscript PDF file). The data should be provided as part of the manuscript or its supporting information, or deposited to a public repository. For example, in addition to summary statistics, the data points behind means, medians and variance measures should be available. If there are restrictions on publicly sharing data—e.g. participant privacy or use of data from a third party—those must be specified.

Reviewer #1: Yes

Reviewer #2: (No Response)

**********

5. Is the manuscript presented in an intelligible fashion and written in standard English?

PLOS ONE does not copyedit accepted manuscripts, so the language in submitted articles must be clear, correct, and unambiguous. Any typographical or grammatical errors should be corrected at revision, so please note any specific errors here.

Reviewer #1: Yes

Reviewer #2: No

**********

6. Review Comments to the Author

Please use the space provided to explain your answers to the questions above. You may also include additional comments for the author, including concerns about dual publication, research ethics, or publication ethics. (Please upload your review as an attachment if it exceeds 20,000 characters)

Reviewer #1: (No Response)

Reviewer #2: In point 10 of the answer to reviewer comments, you argue that further analysis and details of the NGS genome sequencing results will be reoprted "in the next paper"...and added that comment in the text (P29, L566-570). Please change that for a more adequate statement, such as "analysis of NGS data is still ongoing and the results will be published elsewhere"

**********

7. PLOS authors have the option to publish the peer review history of their article (what does this mean?). If published, this will include your full peer review and any attached files.

If you choose “no”, your identity will remain anonymous but your review may still be made public.

**Do you want your identity to be public for this peer review?** For information about this choice, including consent withdrawal, please see our Privacy Policy.

Reviewer #1: Yes: Fatemeh Fotouhi

Reviewer #2: No

[NOTE: If reviewer comments were submitted as an attachment file, they will be attached to this email and accessible via the submission site. Please log into your account, locate the manuscript record, and check for the action link "View Attachments". If this link does not appear, there are no attachment files to be viewed.]

While revising your submission, please upload your figure files to the Preflight Analysis and Conversion Engine (PACE) digital diagnostic tool, https://pacev2.apexcovantage.com/. PACE helps ensure that figures meet PLOS requirements. To use PACE, you must first register as a user. Registration is free. Then, login and navigate to the UPLOAD tab, where you will find detailed instructions on how to use the tool. If you encounter any issues or have any questions when using PACE, please email us at figures@plos.org. Please note that Supporting Information files do not need this step.

# Author comment: Author response to Decision Letter 1

**DOI:** 10.1371/journal.pone.0229601.r004

6 Feb 2020

Response to Academic Editor and Reviewers

Thank you for the letter and for the reviewer’s comment. We would like to report that our MS was revised to meet PLOS ONE’s publication criteria. We respond to each point raised by the academic editor and reviewers during the review process. To comply with your suggestions, we requested a professional English language editing company, Editage, to copyedit thoroughly our manuscript for language usage, clarification on technical expression, spelling, and grammar and any typographical or grammatical errors are corrected at revision to help the readability of the paper. 

Reviewer #2: 

1. In point 10 of the answer to reviewer comments, you argue that further analysis and details of the NGS genome sequencing results will be reported "in the next paper"...and added that comment in the text (P29, L566-570). Please change that for a more adequate statement, such as "analysis of NGS data is still ongoing and the results will be published elsewhere"

Response to comment:

Lines 567-568, page 29: We changed the sentence to be a more adequate statement as you suggested.

Attachment Submitted filename: Response to Reviewers.doc Click here for additional data file.

# Editor report: Decision Letter 2

**DOI:** 10.1371/journal.pone.0229601.r005

**Authors:** Baochuan Lin

11 Feb 2020

Epidemic of influenza A(H1N1)pdm09 analyzed by full genome sequences and the first case of oseltamivir-resistant strain in Myanmar 2017

PONE-D-19-24652R2

Dear Dr. Kyaw Win,

We are pleased to inform you that your manuscript has been judged scientifically suitable for publication and will be formally accepted for publication once it complies with all outstanding technical requirements.

Within one week, you will receive an e-mail containing information on the amendments required prior to publication. When all required modifications have been addressed, you will receive a formal acceptance letter and your manuscript will proceed to our production department and be scheduled for publication.

Shortly after the formal acceptance letter is sent, an invoice for payment will follow. To ensure an efficient production and billing process, please log into Editorial Manager at https://www.editorialmanager.com/pone/, click the "Update My Information" link at the top of the page, and update your user information. If you have any billing related questions, please contact our Author Billing department directly at authorbilling@plos.org.

If your institution or institutions have a press office, please notify them about your upcoming paper to enable them to help maximize its impact. If they will be preparing press materials for this manuscript, you must inform our press team as soon as possible and no later than 48 hours after receiving the formal acceptance. Your manuscript will remain under strict press embargo until 2 pm Eastern Time on the date of publication. For more information, please contact onepress@plos.org.

With kind regards,

Baochuan Lin, Ph.D.

Academic Editor

PLOS ONE

Additional Editor Comments (optional):

Reviewers' comments:

# Editor report: Acceptance letter

**DOI:** 10.1371/journal.pone.0229601.r006

**Authors:** Baochuan Lin

19 Feb 2020

PONE-D-19-24652R2 

Epidemic of influenza A(H1N1)pdm09 analyzed by full genome sequences and the first case of oseltamivir-resistant strain in Myanmar 2017 

Dear Dr. Kyaw Win:

I am pleased to inform you that your manuscript has been deemed suitable for publication in PLOS ONE. Congratulations! Your manuscript is now with our production department. 

If your institution or institutions have a press office, please notify them about your upcoming paper at this point, to enable them to help maximize its impact. If they will be preparing press materials for this manuscript, please inform our press team within the next 48 hours. Your manuscript will remain under strict press embargo until 2 pm Eastern Time on the date of publication. For more information please contact onepress@plos.org.

For any other questions or concerns, please email plosone@plos.org. 

Thank you for submitting your work to PLOS ONE.

With kind regards,

PLOS ONE Editorial Office Staff

on behalf of

Dr. Baochuan Lin 

Academic Editor

PLOS ONE
